# An *in silico* procedure for generating protein-mediated chromatin interaction data and comparison of significant interaction calling methods

**DOI:** 10.1371/journal.pone.0287521

**Published:** 2024-01-17

**Authors:** Shuyuan Lou, Shili Lin

**Affiliations:** 1 Interdisciplinary Ph.D. Program in Biostatistics, The Ohio State University, Columbus, OH, United States of America; 2 Department of Statistics, The Ohio State University, Columbus, OH, United States of America; 3 Translational Data Analytics Institute, The Ohio State University, Columbus, OH, United States of America; Penn State: The Pennsylvania State University, UNITED STATES

## Abstract

The ability to simulate high-throughput data with high fidelity to real experimental data is fundamental for benchmarking methods used to detect true long-range chromatin interactions mediated by a specific protein. Yet, such tools are not currently available. To fill this gap, we develop an in silico experimental procedure, ChIA-Sim, which imitates the experimental procedures that produce real ChIA-PET, Hi-ChIP, or PLAC-seq data. We show the fidelity of ChIA-Sim to real data by using guiding characteristics of several real datasets to generate data using the simulation procedure. We also used ChIA-Sim data to demonstrate the use of our in silico procedure in benchmarking methods for significant interactions analysis by evaluating four methods for significant interaction calling (SIC). In particular, we assessed each method’s performance in terms of correct identification of long-range interactions. We further analyzed four experimental datasets from publicly available databases and shew that the trend of the results are consistent with those seen in data generated from ChIA-Sim. This serves as additional evidence that ChIA-Sim closely resembles data produced from the experimental protocols it models after.

## Introduction

Chromosomal 3D interactions have been studied to investigate genome structures and functions in the past two decades [1–5]. Advances in biological technologies have contributed to deepened understanding of the 3D structure of a genome. The first assay for quantifying the frequency of physical contacts was chromosome conformation capture (3C) [6], a low-throughput method for two pre-specified DNA fragments. A number of 3C-derivatives have been developed, including chromosome conformation capture-on-chip (4C) [7], chromosome conformation capture carbon copy (5C) [8], and Hi-C [9], the last being a high-throughput assay that produces genome-wide contact maps. All these 3C-based methods aim to capture chromatin interactions regardless of the associated proteins in the binding complex.

There are also protein-centric methods targeting genome-wide chromatin interactions mediated by a specific protein of interest. One of the earliest such methods is ChIP-3C [10], also known as ChIP-loop, which combines chromatin immunoprecipitation (ChIP) with 3C to facilitate detection of protein-specific chromatin interactions. The protocol of ChIP-3C for preparing proximal ligation is similar to 3C, including crosslinking and restriction enzyme digestion, but the crosslinked chromatin is first ChIP-enriched before ligation. Nevertheless, it is still a 3C-based method that can only capture the interactions between two specified segments. With the introduction of pair-end tags (PET) sequencing technology, Chromatin Interaction Analysis with PET (ChIA-PET) was developed [11], which provides a comprehensive assay for detecting whole-genome chromatin interactions mediated by a specific protein; for example, ER*α*. ChIA-PET combines ChIP, linker ligation, and PET sequencing, where ChIP is used to identify transcription factor binding sites; linker ligation is used to connect DNA fragments captured by crosslinking; and PET is used to extract linked pairs for sequencing. The protocol of ChIA-PET has been optimized to improve the signal-to-noise ratio [12]. It replaces the restriction enzyme digestion by sonication for chromatin fragmentation as sonication can shake off the non-specific interactions, thereby reducing the background noise level. Recent advances on protein-centric interactions detection follow the principles of in situ Hi-C [13] that performs the ligation step before cell lysis to enhance the signal-to-noise ratio; examples include HiChIP [14] and proximity ligation-assisted ChIP-seq (PLAC-seq) [15]. HiChIP was proposed to improve ChIA-PET by applying ChIP on the in situ Hi-C library. PLAC-seq was introduced in the same year as HiChIP but with slightly different protocols in the library preparation step. Both HiChIP and PLAC-seq are more efficient and accurate than ChIA-PET in that they use fewer cells and output more contact reads.

Current experimental results from ChIA-PET, HiChIP, and PLAC-seq are indispensable resources for understanding micro-scale mechanisms of long-range and spatial gene regulations. It has been found that particular protein signaling and genome structural variation may alter the long-range regulatory mechanism, hence causing certain diseases [16, 17], especially cancer [10, 18–20]. Output from these experimental procedures is a list of long-range interaction frequencies (counts) between pairs of anchors (genomic segments), which may or may not be on the same chromosome (Table 1 provides a partial list from a biological experiment as an example).

**Table 1:** Partial list of output from a ChIA-PET experiment (GSM832464).

Chr_*a*_	Anchor_*a*_	Chr_*b*_	Anchor_*b*_	Freq.
Chr1	839092 – 842325	Chr1	935528 – 939051	3
Chr1	874165 – 879175	Chr1	933340 – 938306	10
Chr10	73997827 – 74008668	Chr10	74031068 – 74039715	22
Chr1	2438340 – 2441360	Chr17	41463653 – 41467707	7

However, despite technological improvements, identified interactions—those listed in Table 1 for instance—are potentially mixed with noise, especially those with small counts. Nevertheless, an interacting pair with a small interaction count is not necessarily indicative of a false pair, as other factors also play a role, making the inference problem challenging. To distill real signals, a number of statistical methods have been developed for analyzing the data to weed out the noise and obtain a curated list of interacting pairs, referred to as significant interactions calling (SIC) [21–24]. Attempts have been made to compare different methods, but most of such comparisons rely entirely on real data, for which the “ground-truth” is unknown. For the few comparisons that do use simulated data, the simulation models are typically contrived and unrealistic, such as the use of a Poisson model for generating count data and treat them as long-range interactions [25]. While most of these methods claim to have better performance in detecting true chromosomal interactions than the rest, a fair simulation study is lacking. In particular, there does not exist any benchmarking data sets with known ground truth.

In this paper, we describe ChIA-Sim, an *in silico* experimental protocol built on a realistic simulation scheme to mimic the *biological* experimental protocols of ChIA-PET, Hi-ChIP, or PLAC-seq, so that data simulated represent realistic specific-protein-mediated long-range interaction data. Since this *in silico* procedure will follow the major steps in real *biological* experimental protocols, we expect data simulated from ChIA-Sim will truly reflect real data and will provide us a tool for carrying out unbiased evaluations of SIC methods.

As an illustration of the usage of ChIA-Sim, we will use data generated from it to compare the performance of four methods for SIC analysis by assessing the pros and cons and their operational characteristics. Since this paper focuses on the ChIA-Sim data generating procedure, our evaluation of the four method is merely serving as an example. There are a number of other existing methods, and methods that become available in the future may also be assessed similarly with known ground truth ChIA-Sim *in sicilo* data. In addition, we will demonstrate the fidelity of the generated data from ChIA-Sim to real ChIA-PET data using key characteristics from several datasets as input to the simulation procedure. We will also analyze these real datasets to substantiate the observations seen in the simulation study regarding the performance characterstics of the four SIC methods, leading to suggestions on the potential of devising improved methods.

## Materials and methods

### ChIA-Sim *in silico* procedure

**Fig 1:**
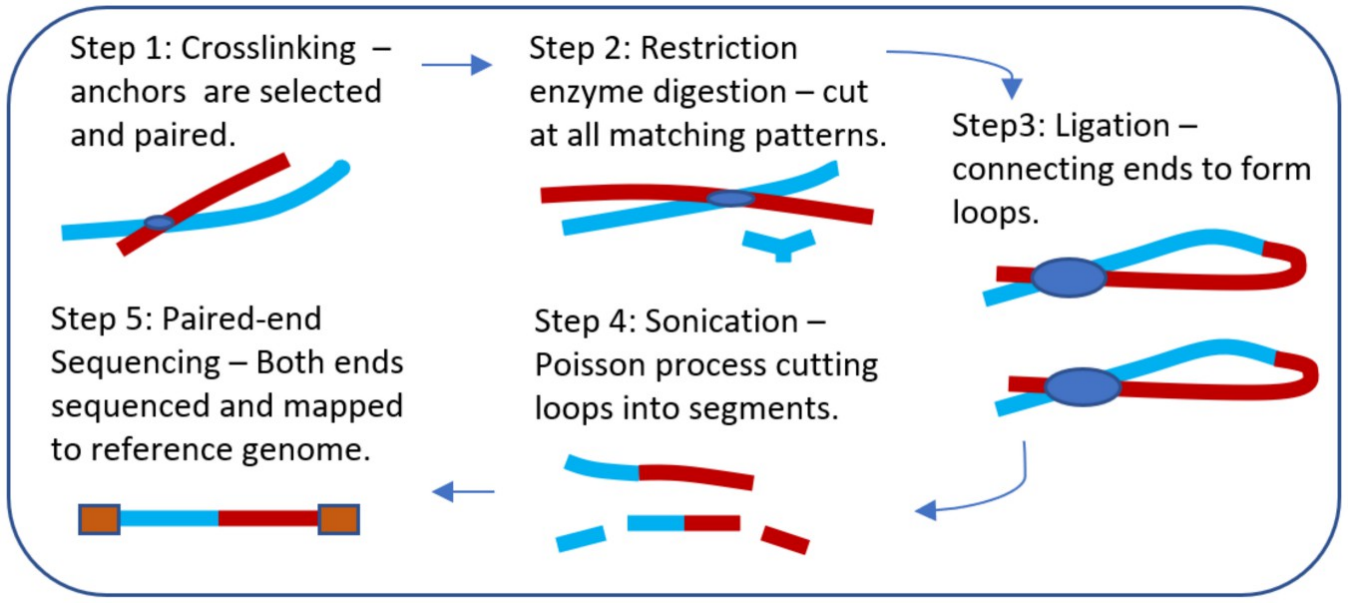
CHIA-Sim *in silico* experimental procedure. The five steps are described in detailed in the text.

In this section, we present the ChIA-Sim *in silico* procedure. The biological experimental assays that are currently available for generating protein-mediated long-range interaction data—ChIA-PET, HiChIP, and PLAC-seq—all consist of five major steps: cross-linking, restriction enzyme digestion, ligation, sonication, and pair-end sequencing. Although the more recent assays, HiChIP [15] and PLAC-seq [14], follow the principles of *in situ* Hi-C [13] to enhance the signal-to-noise ratio, the five steps described in our *in silico* procedure are representative of all three biological experimental procedures. Specifically, in ChIA-Sim, each of these five steps is simulated *in silico* to mimic the steps of the biological experimental procedures (Fig 1), and is described as follows.

*Step 1*: ***Cross-linking***. To imitate the biological experimental step of cross-linking, we first randomly select *N*_*E*_ (set denoted as *E*) and *N*_*P*_ (set denoted as *P*) sites from the known sets of TFBS (enhancer) and TSS (promoter) sites, respectively, where the transcription factor binding sites (TFBS) are determined based on the protein of interest, whereas the transcription start sites (TSS) are obtained from a genome assembly, such as hg19. The reason for this random selection is to reduce the size of the data, if repeated simulations are performed for a large number of times, while maintaining the distribution of TFBS/TSS across chromosomes. However, we note that a user may set *N*_*E*_ and *N*_*P*_ to be the actual numbers in the genome to scale up their simulation. To induce random collisions (noise, non-promoter-enhancer interactions), we also randomly select $N_{\bar{E}}$ (set denoted as $\bar{E}$) and $N_{\bar{P}}$ (set denoted as $\bar{P}$) sites that are neither TFBS nor TSS. Each of the sites in $\left\{E,\bar{E}\right\}$ is paired with each of those in $\left\{P,\bar{P}\right\}$, completing the cross-linking step. In particular, an *E* × *P* pairing is treated as a “true” promoter-enhancer interacting pair, whereas the other pairings are simply “noise,” representing random collisions.

*Step 2*: ***Restriction enzyme digestion***. In the biological experimental protocols, the genome may be cut into fragments using a restriction enzyme. In our *in silico* procedure, we adopt HindIII (AAGCTT) as the default, as it is a frequent choice in real experiments. However, we have designed ChIA-Sim to be flexible so that users may supply the DNA pattern of any desired enzyme. We also note that in the original ChIA-PET protocol, this step was performed via sonication, while more recent assays such as HiChIP and PLAC-seq use restriction enzyme digestion. Therefore, we have also designed ChIA-Sim to provide the “sonication” option by using a Poisson process to randomly break chromosomes into pieces.

*Step 3*: ***Ligation***. To imitate the step in an biological experiment where ligations between one end of each of two different fragments—fixed by cross-linking—take place to form “loops”, we connect one end of a piece from $\left\{E,\bar{E}\right\}$ with a piece from $\left\{P,\bar{P}\right\}$, setting different probabilities, including having different probability for intra-chromosomal pairings (TFBS and TSS sites from the same chromosome) versus inter-chromosomal pairings (TFBS and TSS sites from two different chromosomes) and different probabilities between the *E* × *P* pairings versus the other types of pairings. These probabilities can be used to control the noise level (i.e. proportion of random collisions, and the degrees of difficulty in SIC analysis. Parameter values for controlling these probabilities can be set by a user.

*Step 4*: ***Sonication***. To mimic the sonication (random break) process in ChIA-PET, a Poisson process is applied to each loop formed in the previous step by cutting it into fragments. Specifically, the number of cut sites on a loop is randomly sampled from a Poisson distribution with the mean set to be the length of the loop divided by a typical fragment length—250 bp as the default but it can be changed by a user. Then the locations of the cut sites are sampled independently from a Uniform distribution. The loop is then “cut” into fragments according to the chosen cut sites, and fragments that contain the ligation sites are kept and sequenced in the next step.

*Step 5*: ***Paired-end Sequencing***. Reads of a certain length from the two ends of each kept fragments are obtained and mapped to the reference genome. The default on the read length is set in ChIA-Sim, but it can also take a user’s input. Anchors (peaks of mapped fragments) are identified, and counts of chromatin interactions are recorded.

At the end of these five steps, ChIA-Sim outputs a list of interacting pairs with counts, like those from a real ChIA-PET experiment (Table 1). Two different formatting options are provided and can be converted to one another depending on the need of the user; detailed can be found in the Vignette of the software package, which is available on GitHub (https://github.com/sl-lin/ChIASim.git).

### Methods for SIC analysis

Methods have been developed for SIC analysis of ChIA-PET data. The first such tool is ChIA-PET tool [21] which is a pipeline including a hyper-geometric model for SIC. One shortcoming of ChIA-PET tool is that it only uses interaction counts to make decisions, while other factors that also play critical roles are not taken into account. One such important factor is the genomic distance between the two anchors of an interacting pair, where it has been documented that there is a power-law relationship between interaction frequencies and genomic distances [11, 26]. To address the deficiency of CPT, MICC [22] and Mango [23] were developed to account for the contribution of genomic distance in their decision-making process. On the other hand, DNA segments that are involved in interactions usually involve enhancers, containing TFBS of the protein of interest, and promoters, immediate upstream of TSS. To take such information into consideration, MDM [24] incorporates the distance of the two anchors in a pair to their closest TFBS and TSS. More recently, two pipelines, CID [27] and MACPET [28], were developed for simultaneous peak calling and SIC. The innovations of these two methods focus on the peak calling step, while their SIC analysis make use of existing tools described above: CID uses MICC while MACPET uses Mango. A number of other pipelines have also been developed for processing data generated from HiChIP and PLAC-seq [29–31], although we note that the SIC step in these pipelines are also all similar to those proposed for ChIA-PET data, including the direct use of Mango [31]. From the above discussion, it is clear that the SIC step can be used interchangeably for data generated from ChIA-PET, HiChIP, or PLAC-seq.

Our focus of demonstrating the benchmarking utility of data from ChIA-Sim is on SIC analysis tools; therefore, we just consider the four methods discussed above that proposed novel SIC methods originally proposed for ChIA-PET data (Table 2). Taking the four methods selected as a totality, several factors that are highly informative for SIC analysis are included and highlighted in the table for benchmarking and comparing the methods, although none of the methods possess all the characteristics. First, the marginal count of an anchor (Table 2, Column 1)—the number of all pairings the anchor is involved in—is used by all method. Mango [23] does not consider pairings that are inter-chromosomal, although the existence and biological significance of such interactions have been extensively discussed and documented in the literature (e.g. [11, 21, 22, 24, 32]) and the rest of the methods considered do consider them (Table 2, Column 2). Genomic distance between the two anchors of an interacting pair is also an important feature for SIC analysis, as there may exist a power law governing the inverse relationship between genomic distances and interaction counts [9, 11]: the closer the two anchors in the 1D space the higher the interaction count. Thus, the genomic distance between two anchors can be used as a bias-correction measure for SIC analysis; MICC and Mango incorporate this information into their procedures (Table 2, Column 3). On the other hand, prior information on the particular protein of interest, the salient feature of any protein-mediated assays, offers the opportunity to use genomic annotations TFBS and TSS for distilling true promoter-enhancer interacting pairs [24], and MDM incorporates this information (Table 2, Column 4).

**Table 2:** Characteristics of methods—Ability (✓) to integrate important features in addition to interaction counts data.

Method	Marginal Count	Inter-Chromosomal	Genomic Distance	Genomic Annotation
CPT [21]	✓	✓	X	X
MDM [24]	✓	✓	X	✓
Mango [23]	✓	X	✓	X
MICC [22]	✓	✓	✓	X

In the modeling front, CPT, the first published pipeline for analyzing ChIA-PET data, models the interaction count between a pair of anchors using a hypergeometric distribution that makes use of marginal counts to complete its specification of the parameters of the distribution [21]. SIC inference is determined by calculating the raw, then FDR-adjusted p-values. MDM assumes that the interaction count of an observed pair follows a mixture of two truncated Poisson distributions, with one component modeling the interaction count for true pairs and the other for false pairs due to random collision [24], where the mean interaction frequency of a true pair is assumed to be larger than that of a false pair. Note that the truncation can accommodate the inclusion of only pairs whose interaction counts are above a certain threshold, such as greater than 1, a commonly used data preprocessing strategy [23, 33]. A hierarchical modeling scheme is used for integrating additional data, including marginal counts and genomic annotation on promoters and enhancers, for setting up prior distributions.

For the SIC analysis in Mango [23], there are two preprocessing steps to remove some observed interacting pairs: first, all inter-chromosomal interactions and those intra-chromosomal interactions with the genetic distance between the pair greater than a user-defined value (default: 1Mb); then, those that are determined to be of self-ligations. Binomial distribution is then used to model the interactions between two anchors for the remaining pairs. The probability of an interaction between a pair of anchors is estimated based on the genomic distance between them and the marginal counts. Based on raw p-values, family-wise error rates are obtained using FDR control. MICC [22] also models the observed counts using a mixture of distributions as in MDM, but further divides false interactions into two components, random collision and random ligation, where a random collision is assumed to be independent, while a random ligation is assumed to be dependent on the genomic distance between the two anchors. For counts from true pairs or random collision, the probability of an observed interaction is assumed to be inversely related to the genetic distance of the anchors, whereas the probability of an observed interaction due to random ligation is assumed to follow a hypergeometric distribution using the marginal counts similar to that of CPT. Model parameters are estimated using the Expectation-Maximization algorithm [34] and FDR control is applied to obtain family-wise error rates.

## Results

### Fidelity of ChIA-Sim to real experimental data

We demonstrate, in this subsection, how one may use real ChIA-PET data to provide guidance for ChIA-Sim so that the simulated data will resemble key characteristics of the real data it models after. First, three datasets were generated using ChIA-Sim following the characteristics of a K562 Pol2 ChIA-PET dataset (accession #: GSM832464). There are a total of 879, 264 interactions in the K562 Pol2 real data, which was used as the sequencing depth parameter *N* in ChIA-Sim. Among them, 1,792 pairs were commonly detected as true pairings by all four methods, CPT [21], MDM [24], MICC [22], and Mango [23], which were then taken as representing true interactions. For each pair, we found the nearest pair of promoter and enhancer to the two anchors. That is, for each of these 1,792 pairs, we identified the corresponding TFBS and TSS—for TSS, we used hg19 (GRCh37); for TFBS, we used a ChipSeq dataset (accession #: GSM935475) that contains TFBS targeting Pol2. The aggregate of these enhancers and promoters among all the pairs are then used as *N*_*E*_ and *N*_*P*_, and they are 1359 and 1492, respectively. We then randomly selected 100 non-TSS and 100 non-TFBS loci; i.e. $N_{\bar{P}}=N_{\bar{E}}=100.$ We considered two restriction enzymes, Mmel (scenarios A) and HindIII (scenario B), with the latter frequently used in long-range interaction experiments including Hi-C data [9] while the former used in the K562 Pol2 data we are modeling after. For the simulation using Mmel as the restriction enzyme, we further consider a larger set of potential false pairs by setting $N_{\bar{P}}=N_{\bar{E}}=400$ (scenario C). A summary of these three scenarios are provided in the left block of S1 Table in S1 File.

**Fig 2:**
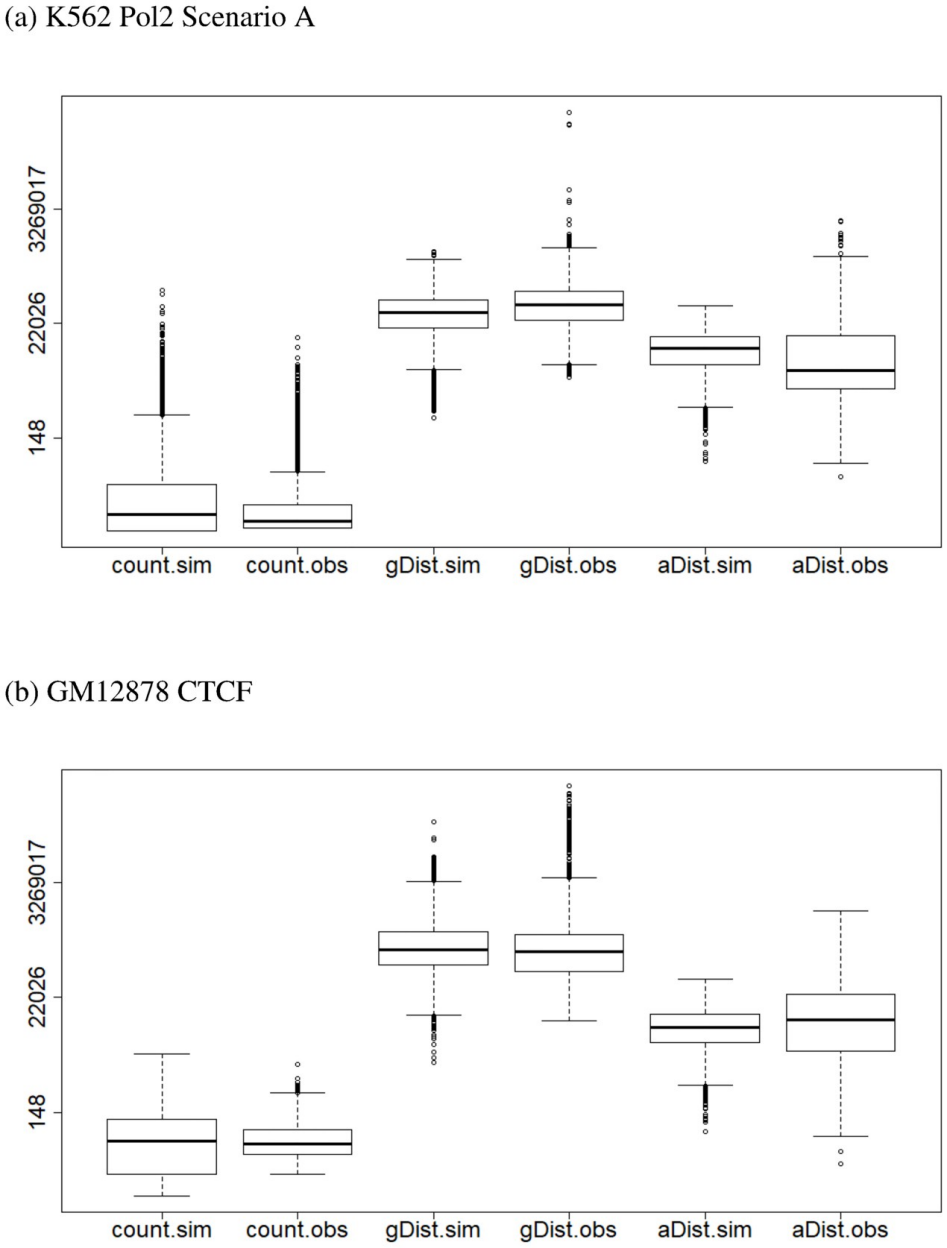
Boxplots comparing several characteristics of data generated from ChIA-Sim with those of the real data based on which the simulation was modeled, for two real datasets. The y-axis is marked on the log-scale: the first two boxplots denotes the interaction counts; the last four boxes denote two types of genomic distances as described in the paper, gDist and aDist. The labels with.obs indicate observed characteristics in the real data, whereas those with.sim presents the corresponding characteristics in the data generated from ChIA-Sim. (a) K562 Pol 2 Scenario A, (b) GM12878 CFCF; the specific parameter settings are provided in S1 Table in S1 File.

The result for scenario A, with Mmel as the restriction enzyme, the same as the real data, are presented in Fig 2(a), while those for scenarios B and C are presented as S1 Fig in S1 File. As can be seen from the figures, the distribution of the simulated counts (first boxplot) has similar profile to that of the observed counts (second boxplot) for all scenarios: both distributions are right skewed, with some large counts; the bottom 75% for both distributions cover a small range in the spectrum of counts. We then examined the genomic distance between the two locations of each pair of anchors (gDist), and found that the the middle 50% of the distances for the simulated data are consistent with those for the observed data (two middle boxplots). Resemblance between the simulated and the real data for another characteristic, the annotation distance (aDist) between the locations of each pair of anchors and the nearest pair of enhancer-promoter, is also observed (last two boxplots). Treating each boxplot in Fig 2(a) as representing a population, Wilcoxon rank-sum test was performed on 100 randomly selected observations from each of the populations to compare the counts, gDist, and aDist between the observed and the simulated distributions, with resulting p-values 0.92, 0.44, and 0.06, respectively.

These results demonstrate the fidelity of the simulated data from ChIA-Sim to the real data based on which the model parameters are set. In addition, the similarity between the results in all scenarios, A, B and C, indicates that setting different numbers of random sites, $N_{\bar{P}}$ and $N_{\bar{E}}$, or using different restriction enzymes, HindIII or Mmel, has little influence on the important characteristics of the simulated data.

To further demonstrate the wider applicability of ChIA-Sim, we also generated data modeled after three other datasets: K562 CTCF (accession #: GSM970216), MCF7 CTCF (accession #: GSM970215), and GM12878 CTCF (accession #: GSM1872886). Compared to the K562 Pol2 data, the transcription factor CTCF binds more distally from gene promoters, and it is a highly conserved transcription factor that has significant implications in spatial gene regulation [35]. Taking GM12878, a lymphobastoid cell line, as an example, we generated interaction counts using the parameters provided in S1 Table in S1 File (last column).

As can be seen from the boxplots (Fig 2(b)), the distributions for the simulated counts (first boxplot), the genomic distance gDist (third boxplot), and the the annotation distance aDist (fifth boxplot), all resemble their observed counterparts (second, fourth, and sixth boxplots, respectively). Note that the simulated counts were scaled so that the total counts are the same as the observed data. The Wilcoxon rank-sum test led to p-values of 0.45, 0.11, and 0.38 for the counts, gDist, and aDist, respectively. The boxplots for the other other two datasets, both with the CTCF transcription factor, are provided in S2 Fig in S1 File, which also show similar key characteristics between the simulated and the observed data.

### An example usage of ChIA-Sim

In this section, we demonstrate the usage of ChIA-Sim for producing benchmarking data to evaluate and compare four methods for SIC analysis: CPT [21], MDM [24], MICC [22], and Mango [23].

#### Setting

We simulated datasets varying several parameters, including “sequencing depth” *N*, “number of true TSS loci” *N*_*P*_ (transcription start sites, mimicking anchors in promoter regions), “number of true TFBS loci” *N*_*E*_ (transcription factor binding sites, mimicking enhancer anchors), “number of non-TSS loci” $N_{\bar{P}}$ (mimicking anchors involving in false pairings), and “number of non-TFBS loci” $N_{\bar{E}}$ (also mimicking anchors involving in false pairings). In particular, *N* is an important parameter controlling the “sequencing depth,” whereas the other four parameters are involved in controlling the “noise” level: the larger the *N*_*P*_ and *N*_*E*_ relative to $N_{\bar{P}}$ and $N_{\bar{E}}$, the smaller the noise level. The ratio between intra- and inter-chromosomal interactions may also be controlled by several other runtime parameters. Further, proportions of “true” interactions to “false” interactions (involving at least one non-TFBS or non-TSS anchor) may also be controlled. In this simulation, we set all “false” interactions to be between non-TFBS and non-TSS anchors. Among the “true” loops, one may also control the degree of “easiness” so that some loops are easier to be inferred correctly in the SIC analysis while others are more difficult. Specifically, to investigate the effect of sequencing depth, we considered four settings where *N* is set to be 1 × 10^5^, 3 × 10^5^, 6 × 10^5^, and 1 × 10^6^, labelled as settings 1, 2, 3, and 4, respectively, whereas $N_{P}=N_{E}=N_{\bar{P}}=N_{\bar{E}}=400$. We also considered another setting, setting 5, where *N* = 1 × 10^6^ and $N_{P}=N_{E}=N_{\bar{P}}=N_{\bar{E}}=1000$ to study the effect of a larger number of sites. In addition, we considered one more setting, setting 6, where *N* = 1 × 10^6^, $N_{P}=1500,N_{E}=500,N_{\bar{P}}=N_{\bar{E}}=400$ (S2 Table in S1 File). This last setting was chosen as a representative to reflect the relative proportions of three types of sites for the three datasets with CTCF as the transcription factor: enhancer, promoter, and non-specific binding sites (S3 Table in S1 File), where the ratio of the number of enhancers to that of promoters ranges from 3 to 15 while the ratio of promoters to non-specific binding sites ranges from 0.4 to 49. As an aside, we note that there is a large difference between CTCF and Pol2, not only for the relative proportions of the three types of sites, but also for the types of pairs (S4 Table in S1 File).

We manipulated the ligation probabilities so that a true pairing between *P* and *E* is nine times more likely than the false pairing between $\bar{P}$ and $\bar{E}$ (random collisions). For each of the settings, we generated five replicates to gauge the consistency of results within each of the SIC methods being compared. For a thorough investigation, in addition to the originally generated datasets from ChIA-Sim, we also created corresponding datasets which only retain pairs that interact more than once (i.e. pairs with count = 1 in the list produced by ChIA-Sim are removed), as this is a common practice of data preprocessing in a number of SIC methods [21]. For ease of reference, datasets that have interacting counts greater than 1 are call the g1 datasets; for example, a dataset from setting 2g1 refers to one that was simulated under setting 2 but only keeping interacting counts that are greater than 1.

#### Comparison of four methods

The results from applying four methods, CPT, MICC, MDM, and Mango, to the simulated data sets retaining counts greater than one (the g1 datasets) are provided in Table 3, where the false positive rate (FPR; identification of false loops due to random pairing) and power (identification of true loops due to long-range interactions) are shown. The results for those with all pairs (i.e. including pairs that only interacted once) are relegated to Supplementary S5 Table in S1 File, as we observe similar trends. We first note that CPT [21] and MICC [22] software packages do not provide a default threshold for decision making. Hence, we used an FDR of 0.05 for MICC as it was also used in their own study [22]; we used an FDR of 0.01 for CPT considering the FDRs from CPT were all very small in our results. On the other hand, both the MDM [24] and Mango [23] packages provide a default decision rule: MDM declares pairs with a posterior probability greater than 0.5 as true interacting; whereas Mango identifies those with FDR less than 0.05 and having interacting counts of at least 2 as true pairs. These default decision rules were used in our analysis. In Table 3 and S5 Table in S1 File, overall FPR and power, as well as their breakdown into intra- and inter-pairs, are provided as the mean and standard error (those in parentheses) over five replicates of our simulated data. As can be seen from the tables, CPT labeled almost all pairs as significant. For MICC, it shows good power, as high as 0.9 (for setting 4g1), but with unacceptably high FPRs, as high as 0.8 (for setting 4). Note that there were no results provided for MICC in settings 5 and 6 due to errors when running the software. Mango, on the other hand, cannot deal with inter-chromosomal pairs; hence, results are available only for intra pairs. We observe that it also has severely inflated FPRs, but generally with lower power than MICC or CPT. On the other hand, MDM has the lowest power but its FPR is very well controlled, albeit a bit conservative. In summary, across all the settings and replicates, CPT, MICC, and Mango all have higher power than MDM, but the higher power is offset by their severely inflated false positive rates. These results are consistent from replicate to replicate.

**Table 3:** Simulation results for pairs with counts > 1. The average and standard deviation (within parentheses) across five replicates are provided for each setting and each method.

Setting	Type	Criteria	CPT	MDM	MICC	Mango
1g1	Overall	FPR	1.000 (0.000)	0.024 (0.034)	0.166 (0.041)	- (-)
		power	0.984 (0.011)	0.244 (0.043)	0.715 (0.042)	- (-)
	Intra	FPR	1.000 (0.000)	0.024 (0.034)	0.167 (0.041)	0.685 (0.098)
		power	0.985 (0.011)	0.261 (0.046)	0.732 (0.041)	0.753 (0.040)
	Inter	FPR	1.000 (0.000)	0.000 (0.000)	0.000 (0.000)	- (-)
		power	0.976 (0.018)	0.011 (0.009)	0.477 (0.072)	- (-)
2g1	Overall	FPR	0.999 (0.002)	0.006 (0.010)	0.272 (0.053)	- (-)
		power	0.989 (0.010)	0.317 (0.078)	0.813 (0.063)	- (-)
	Intra	FPR	0.999 (0.002)	0.006 (0.010)	0.278 (0.054)	0.836 (0.228)
		power	0.991 (0.010)	0.349 (0.087)	0.835 (0.060)	0.819 (0.055)
	Inter	FPR	1.000 (0.000)	0.000 (0.000)	0.065 (0.058)	- (-)
		power	0.976 (0.022)	0.024 (0.022)	0.600 (0.113)	- (-)
3g1	Overall	FPR	0.999 (0.001)	0.008 (0.005)	0.326 (0.060)	- (-)
		power	0.996 (0.003)	0.322 (0.066)	0.849 (0.034)	- (-)
	Intra	FPR	0.999 (0.001)	0.009 (0.005)	0.337 (0.062)	0.966 (0.043)
		power	0.996 (0.003)	0.355 (0.072)	0.864 (0.031)	0.854 (0.038)
	Inter	FPR	1.000 (0.000)	0.000 (0.000)	0.087 (0.028)	- (-)
		power	0.994 (0.007)	0.025 (0.025)	0.715 (0.074)	- (-)
4g1	Overall	FPR	1.000 (0.000)	0.001 (0.001)	0.306 (0.103)	- (-)
		power	0.998 (0.001)	0.484 (0.067)	0.897 (0.017)	- (-)
	Intra	FPR	1.000 (0.000)	0.001 (0.001)	0.321 (0.108)	0.945 (0.049)
		power	0.998 (0.001)	0.528 (0.069)	0.911 (0.016)	0.847 (0.019)
	Inter	FPR	0.999 (0.002)	0.000 (0.000)	0.088 (0.058)	- (-)
		power	0.999 (0.003)	0.105 (0.061)	0.774 (0.064)	- (-)
5g1	Overall	FPR	0.999 (0.001)	0.017 (0.004)	- (-)	- (-)
		power	0.976 (0.013)	0.185 (0.095)	- (-)	- (-)
	Intra	FPR	0.999 (0.001)	0.018 (0.004)	- (-)	0.929 (0.122)
		power	0.977 (0.012)	0.204 (0.106)	- (-)	0.872 (0.040)
	Inter	FPR	0.999 (0.001)	0.000 (0.000)	- (-)	- (-)
		power	0.967 (0.019)	0.004 (0.009)	- (-)	- (-)
6g1	Overall	FPR	0.994 (0.001)	0.026 (0.001)	- (-)	- (-)
		power	0.972 (0.001)	0.121 (0.003)	- (-)	- (-)
	Intra	FPR	0.993 (0.001)	0.028 (0.001)	- (-)	0.978 (0.002)
		power	0.973 (0.001)	0.133 (0.003)	- (-)	0.913 (0.004)
	Inter	FPR	0.999 (0.002)	0.000 (0.000)	- (-)	- (-)
		power	0.957 (0.003)	0.001 (0.000)	- (-)	- (-)

**Fig 3:**
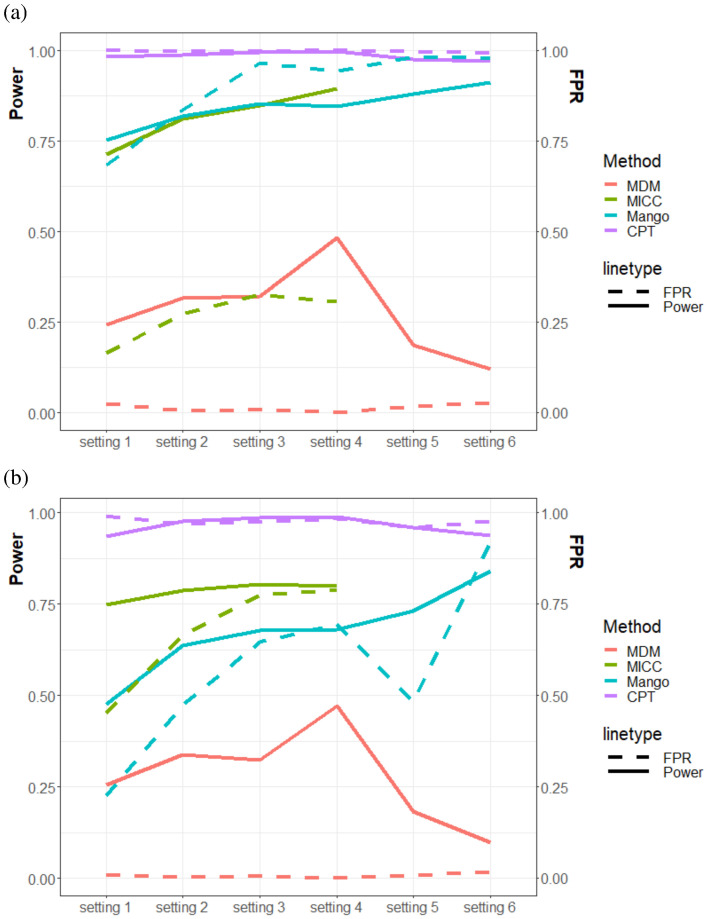
Comparison of methods based on mean power (left axis) and mean FPR (right axis). Each line is the average power/FPR over the 5 replicates of each setting. (a) Datasets consisting of pairs with interaction counts greater than 1; (b) All pairs. No results were plotted for MICC with settings 5 and 6 due to errors when running the software.

For a better visualization of the results, and especially for understanding the effects of *N* on the outcomes, we plotted the mean power and mean FPR across the five replicates for each setting in Fig 3. First, we see that power generally increases when *N* increases (from setting 1 to setting 4, with or without including pairs that interact only once). Unfortunately, the FPR also increases with the increase of *N*, except for MDM where the FPR are always very low. On the other hand, when *N* is set to be the same but with either increasing or uneven numbers of sites (from setting 4 to setting 5 or setting 6), both CPT and MDM see a decrease in power regardless of whether the one-interacting pairs were included, whereas Mango sees an increase in power. It is interesting to observe that Mango has a steep decrease in FPR for setting 5, which is explainable by the greater occurrences of one-interacting pairs and such pairs, more likely to represent false loops, are already removed in Mango’s preprecessing steps. However, its FPR increases sharply for setting 6, which is in fact higher than its power. On the other hand, MICC was unsuccessful in obtaining results for the data in both settings 5 and 6 due to errors when running the software.

**Fig 4:**
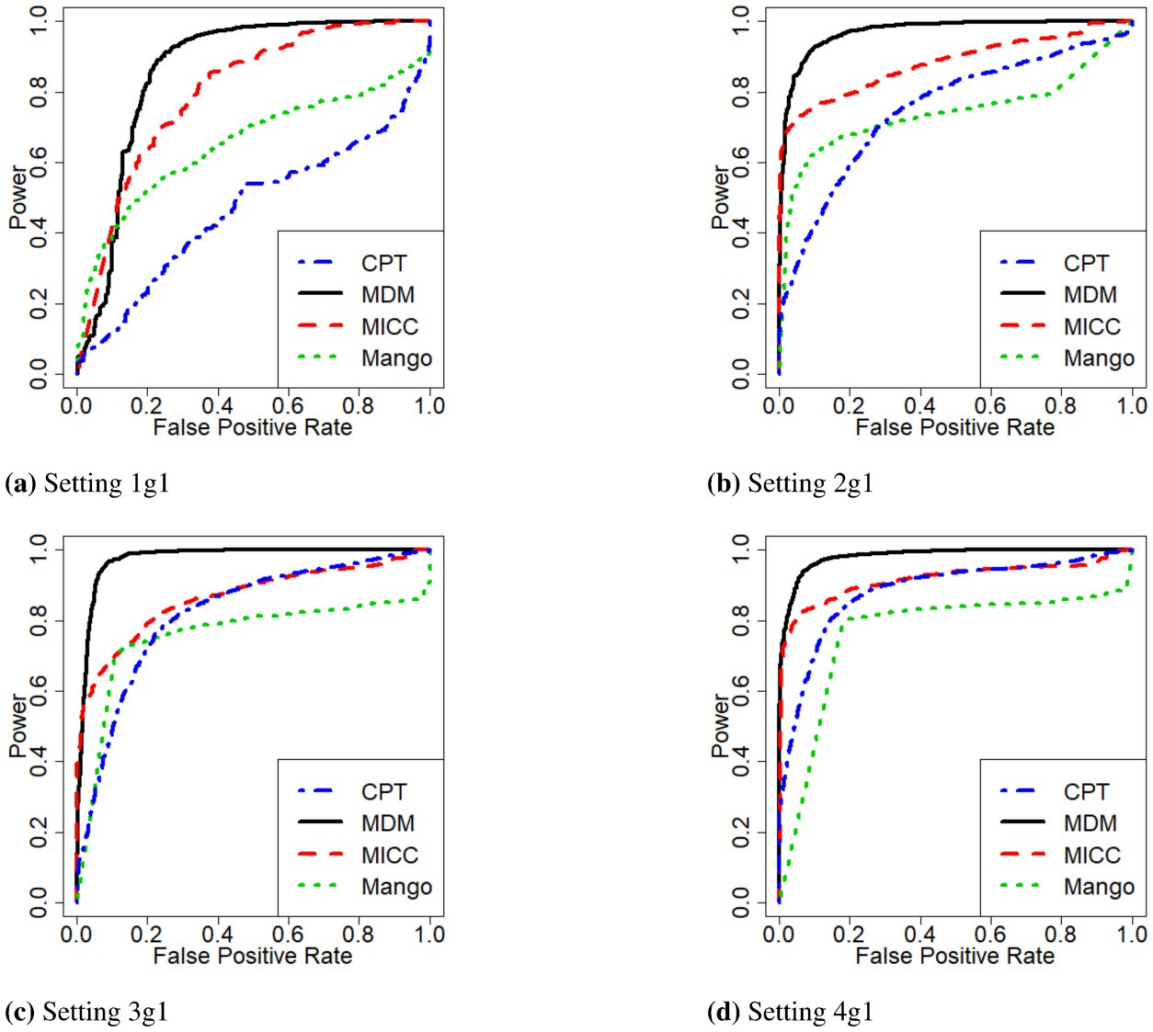
Receiver operating characteristic (ROC) curves for comparing the methods—g1 pairs of replicate 1.

To provide more informative results and a fairer comparison among the methods, we obtain the power of the methods for each given false positive rate, and the result for replicate 1 for a range of false positive rates (plotted on the x-axis) are shown in Fig 4 and S3 Fig in S1 File for g1 pairs and all pairs, respectively, for the first four settings where results are available for all four methods. We can see that MDM appears to have the best performance overall, with much larger area under the curve (AUC) values for both g1 pairs (Table 4) and all pairs (S6 Table in S1 File) across all replicates. Nevertheless, we note that MICC and Mango can have higher power for very small FPRs for the scenario with g1 pairs (Fig 4). Further, CPT or Mango are seen to be generally inferior to MICC or MDM for the g1 data. However, for the data including all pairs, other than setting 1, MICC’s performance appears to be lagging.

**Table 4:** Performance evaluation based on AUC for pairs with counts > 1.

Setting	Replicate	CPT	MDM	MICC	Mango
1g1	1	0.469	0.860	0.801	0.654
	2	0.570	0.976	0.868	0.706
	3	0.580	0.970	0.841	0.697
	4	0.536	0.952	0.859	0.633
	5	0.504	0.927	0.839	0.670
2g1	1	0.753	0.970	0.882	0.742
	2	0.655	0.927	0.754	0.676
	3	0.759	0.981	0.871	0.702
	4	0.779	0.983	0.882	0.742
	5	0.741	0.985	0.884	0.748
3g1	1	0.821	0.975	0.865	0.758
	2	0.743	0.958	0.809	0.688
	3	0.863	0.996	0.922	0.715
	4	0.743	0.965	0.838	0.715
	5	0.775	0.965	0.828	0.742
4g1	1	0.884	0.982	0.916	0.765
	2	0.849	0.985	0.878	0.707
	3	0.812	0.982	0.885	0.673
	4	0.876	0.994	0.911	0.786
	5	0.870	0.994	0.934	0.726
5g1	1	0.802	0.967	-	0.723
	2	0.607	0.934	-	0.678
	3	0.603	0.946	-	0.691
	4	0.625	0.944	-	0.721
	5	0.599	0.940	-	0.684
6g1	1	0.555	0.906	-	0.538
	2	0.558	0.906	-	0.541
	3	0.563	0.907	-	0.55
	4	0.556	0.907	-	0.539
	5	0.561	0.907	-	0.537

Results for all replicates are consistent with the above discussion and are provided in S4 and S5 Figs in S1 File, which also include those for settings 5 and 6 for completeness, even though there were no results for MICC.

### Analyses of experimental datasets

To further substantiate the observations of the operating characteristics of the four SIC analysis methods, we applied the same four methods to several experimental datasets, K562 Pol2, K562 CTCF, MCF7 CTCF, and GM12878 CTCF, the same four datasets used for our fidelity study in Section 3.1. For K562 Pol2, in addition to the all and g1 pairs as we did in the simulation study, we also consider the g2 pairs (i.e. removing pairs with one and two interaction counts), which may be viewed as more reliable so that we can more thoroughly compare the four methods. For K562 CTCF and MCF7 CTCF, we analyzed the g1 pairs, as these data were already filtered in the original study. For GM12878 CTCF, we analyzed g3 pairs, as pairs with at most three interaction counts were already removed in the available data. Throughout all the analyses, the significance calling thresholds for all the methods are the same as in the simulation study, but we also used the same FDR of 0.05 for CPT as for MICC to illustrate that setting a smaller FDR threshold for CPT did not change the overall conclusion. In the following, we describe, in detail, the results for K562 Pol2. The results for the other datasets show the same pattern and thus are qualitatively the same as those for K562 Pol2; therefore, they will be summarized together, with details given in the Supplementary.

We obtained the K562 Pol2 ChIA-PET data from the ChiaSig package [25]. This dataset contains intra-chromosomal interactions only, which is a subset of the original dataset that also contains inter-chromosomal pairs. We chose to restrict our analysis to this subset that only contains intra-chromosomal interactions since Mango can only handle intra-chromosomal data, and this provided a fairer comparison among the methods. One can see that MICC identifies 25.1, 47.4, and 53% of all, g1, and g2 pairs, respectively, as true interacting pairs, where the increase in the proportion reflects the increase in the reliability of the remaining pairs with a more and more stringent filtering criterion, although the increase from all to g1 is much larger (Table 5). For MDM, 11.3, 18.6, and 17.1% of all, g1, and g2 pairs, respectively, are called as true interacting pairs, with the trend similar to that of MICC—a large increase from all to g1 but almost the same, even a slight decrease, from g1 to g2. Despite the discrepancy in the number and proportions of true pairs identified, MICC and MDM have considerable agreement with each other: they agree on 80.3, 65.4, and 61.8% of callings for all, g1, and g2 pairs (Tables 5 and 6). For CPT, it called 83.7, 98.1, and 99.4% of all, g1, and g2 pairs as truly interacting, obviously an inflation but not as a surprise, since the result is qualitatively the same as that in the simulation study. Finally, Mango identified 1.2, 7.9, and 25% of all, g1, and g2 pairs as true interactions. Although Mango has at least a 68% agreement with MDM and 44% with MICC, we caution that these large agreements are due to the fact that most of the pairs are labelled false pairs by Mango. CPT also has a large agreement with MICC, especially for the g2 pairs. However, note that CPT calls practically 100% of the pairs as true interaction; therefore, these large agreements simply reflect the proportions of true pair callings by MICC.

**Table 5:** K562 Pol2: Percent of identified true pairs and pairwise agreement over total number of pairs^1^.

(a) all pairs^2^				
	CPT	MDM	MICC	True
CPT				83.7
MDM	25.6 (10.3, 15.3)			11.3
MICC	31.8 (20.3, 11.5)	80.3 (8.3, 72.0)		25.1
Mango	17.5 (1.2, 16.3)	88.8 (0.6, 88.2)	75.2 (0.7, 74.5)	1.2
(b) g1 pairs^2^				
	CPT	MDM	MICC	True
CPT				98.1
MDM	20.2 (18.4, 1.7))			18.6
MICC	45.5 (45.5, 0.0)	65.4 (15.7, 49.7)		47.4
Mango	9.7 (7.9, 1.8)	80.4 (3.5, 77.0)	53.5 (4.4, 49.1)	7.9
(c) g2 pairs^2^				
	CPT	MDM	MICC	True
CPT				99.4
MDM	17.6 (17.0, 0.6)			17.1
MICC	52.7 (52.7, 0.0)	61.8 (16.1, 45.7)		53.3
Mango	24.6 (24.5, 0.1)	68.8 (5.4, 63.3)	44.3 (11.3, 33.0)	25.0
(d) g2 pairs^3^				
	CPT	MDM	MICC	True
CPT				99.7
MDM	17.3 (17.0, 0.3)			17.1
MICC	53.0 (53.0, 0.0)	61.8 (16.1, 45.7)		53.3
Mango	24.8 (24.7, 0.0)	68.8 (5.4, 63.3)	44.3 (11.3, 33.0)	25.0

^1^The last column (“True”) is the percent of identified true pairs by a method, while the two percentages in each pair of parentheses are agreements on true and false, respectively.

^2^FDR threshold for CTP is 0.01.

^3^FDR threshold for CTP is 0.05.

**Table 6:** K562 Pol2: Cross-tabulation of the results for four methods^1^.

(a) all pairs^2^							
		MICC	T	T	F	F	
CPT	Mango	MDM	T	F	T	F	Total
T	T		739	213	119	422	1493
T	F		9753	15657	2718	78874	107002
F	T		0	0	0	0	0
F	F		308	5827	969	13986	21090
		Total	10800	21697	3806	93282	129585
(b) g1 pairs^2^							
		MICC	T	T	F	F	
CPT	Mango	MDM	T	F	T	F	Total
T	T		1082	780	393	1123	3378
T	F		5594	12066	845	20217	38722
F	T		8	3	0	0	11
F	F		52	740	0	0	792
		Total	6736	13589	1238	21340	42903
(c) g2 pairs^2^							
		MICC	T	T	F	F	
CPT	Mango	MDM	T	F	T	F	Total
T	T		1259	1559	153	3445	6416
T	F		2941	8026	105	8525	19597
F	T		10	126	0	0	136
F	F		0	28	0	0	28
		Total	4210	9739	258	11970	26177
(d) g2 pairs^3^							
		MICC	T	T	F	F	
CPT	Mango	MDM	T	F	T	F	Total
T	T		1263	1615	153	3445	6476
T	F		2941	8045	105	8525	19616
F	T		6	70	0	0	76
F	F		0	9	0	0	9
		Total	4210	9739	258	11970	26177

^1^The figure provided in each cell within the table is the number of pairs that are agreed or disagreed among four methods.

^2^FDR threshold for CTP is 0.01, which correspond to (a)-(c) in Table 5.

^3^FDR threshold for CTP is 0.05, which corresponds to (d) in Table 5.

Finally, we see that the results for using an FDR threshold of 0.05 for CPT with the g2 pairs data are practically the same as those using an FDR threshold of 0.01 (Tables 5(c), 5(d) and 6(c), 6(d)), because almost all pairs are labelled as true interacting pairs using both thresholds. Taken together, the large agreement of these results from analyzing a real dataset with those based on the ChIA-Sim data is a further indication that our ChIA-Sim *in-silico* procedure indeed mimics experimental ChIA-PET protocol.

For the three datasets with CTCF as the transcription factor, the results are given in S7–S9 Tables in S1 File. As extreme as with the K562 Pol2 data, CPT identified all pairs as true interacting pairs. For MDM and MICC, a moderate proportion of pairs are identified as true interactions, with MICC typically identifies a large proportion than MDM, especially for the GM12879 data, consistent with the K562 Pol2 results. MICC and MDM also have a large proportion of agreement among all three datasets. The results for Mango, on the other hand, depart markedly from that for the K562 Pol2 data, in that almost all pairs were identified as true interacting pairs with the CTCF transcription factor. For visual comparison of the performance between the real data and the simulated ones, we created ROC plots for the four experimental data, treating enhancer-promoter and enhancer-enhancer interactions as “true positives” and the rest as “false positives”. We included the enhancer-enhancer pairs as such pairings are biologically meaningful in forming protein complex for regulating gene expressions. It is interesting to see that, for the three datasets with CTCF as the transcription factor, the curves are very similar to those from the simulation study. However, for K562 Pol2, the result is a bit different from the rest, most notable being that the AUC for CPT is less than 0.5 (S6 Fig in S1 File).

**Fig 5:**
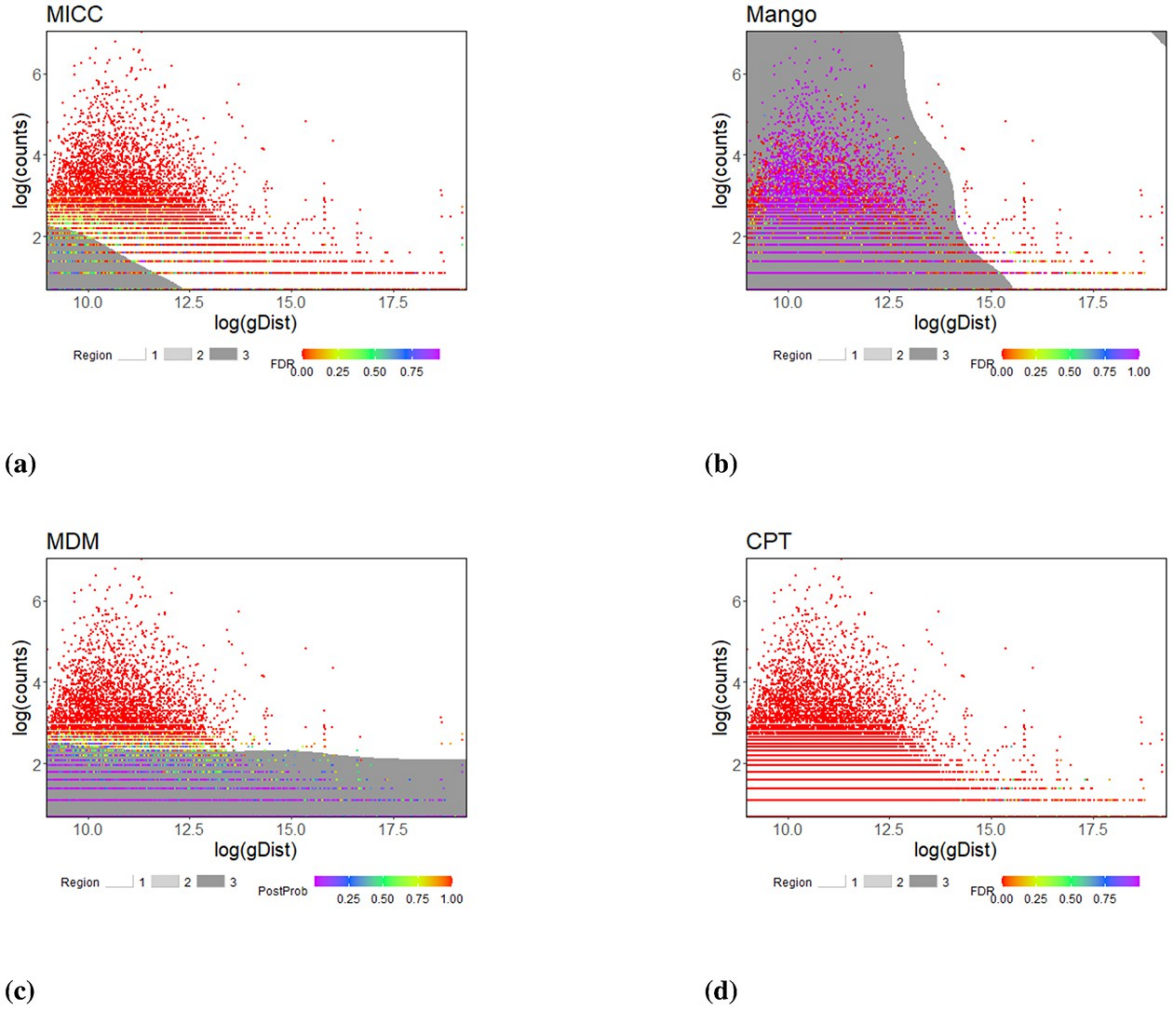
Scatterplot of log(gDist) vs. log(count), with the color gradient of the dots indicating FDR (for MICC, Mango, and CTP) or posterior probability (PP, for MDM). Points in each of the plot are divided into three groups: (1) significant—FDR (0, 0.05) or PP (0.8, 1); (2) moderately-significant—FDR (0.05, 0.2) or PP (0.5, 0.8); (3) non-significant—FDR (0.2, 1) or PP (0, 0.5). The decision boundaries are generated by support vector machine using the R-package e1071 [36], leading to regions shaded in white (Region 1: significant), light-grey (Region 2: moderately significant), and dark grey (Region 3: non-significant), respectively.

**Fig 6:**
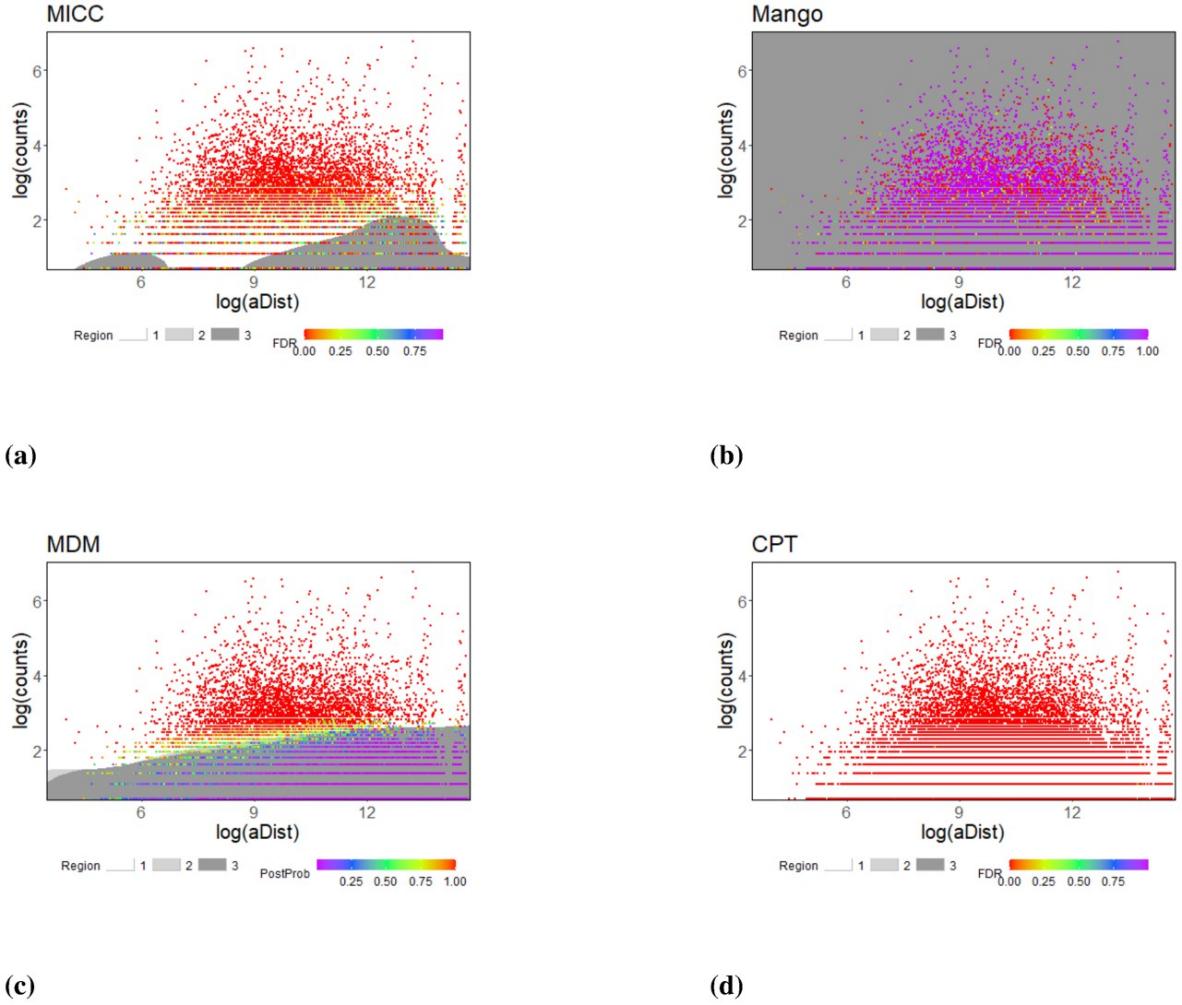
Scatterplot of log(aDist) vs. log(count), with the color gradient of the dots indicating FDR (for MICC, Mango, and CTP) or posterior probability (PP, for MDM). Points in each of the plot are divided into three groups: (1) significant—FDR (0, 0.05) or PP (0.8, 1); (2) moderately-significant—FDR (0.05, 0.2) or PP (0.5, 0.8); (3) non-significant—FDR (0.2, 1) or PP (0, 0.5). The decision boundaries are generated by support vector machine using the R-package e1071 [36], leading to regions shaded in white (Region 1: significant), light-grey (Region 2: moderately significant), and dark grey (Region 3: non-significant), respectively.

Similar results between the simulation study and real data analysis notwithstanding, none of the methods utilize both important features in generating experimental ChIA-PET type data that can affect the performance of the results, namely, the gDist, the genomic distance between each pair of anchors, and the aDist, the genomic distancs from a pair of anchors to the nearest pair of promoter-enhancer. To understand how the results from each of the methods reflect their dependency on these two types of distances, for the K562 Pol2 data, we plotted interaction counts against gDist (Fig 5) and aDist (Fig 6), all on the log-scale, for the analysis results with g1 pairs. The scatter plots are superimposed by support vector machine (SVM) generated decision boundaries: dark grey, light grey, and white regions indicating non-significant, moderate-significant, and significant regions, respectively. That is, the SVM boundaries divide the plane into three regions. The white region contains pairs that are labelled as true interacting pairs (either small false discovery rate or large posterior probability); the dark grey region consists of pairs that have no evidence of being signifying true interactions (either with large false discovery rate or with small posterior probability); the light grey region is composed of the rest of the pairs that have some evidence of being a true pairs (moderately small false discovery rate or moderately large posterior probability). The light grey region is usually difficult to see because it is small (e.g. the MDM plot in Fig 6 shows a visible but small light-grey region); therefore, even though the SVM attempted to separate each plane into three regions, the middle one is typically very small, indicating that, for most pairs, there are strong evidence for them to be either a true interaction pair or not.

For MICC and Mango, which take gDist into account for inference, the results are as expected: since pairs with smaller gDist are easier to be randomly ligated in the experiment, they need a greater interaction count to be declared a true pair; likewise, when two anchors are far apart, that is, with a large gDist, we expect pairs with relatively fewer interaction counts to be identified as positive. This is evident in the sharp fall of the decision boundary for the pairs declared to be non-significant (Fig 5(a) and 5(b)). On the other hand, the decision boundaries for MDM are basically flat, which is also expected since it does not use gDist for inference (Fig 5(c)). For CPT, almost all pairs are classified as significance; hence, the decision boundaries are not visually apparent, and thus not gDist dependent, which is also not surprising given the procedure does not make use of gDist either. In contrast, except for MDM, none of the other methods show a systematic trend according to aDist in the decision boundaries (Fig 6(a), 6(b) and 6(d)). The gradual increase in the boundaries of MDM (Fig 6(c)) are anticipated as it assigns higher posterior probabilities to pairs that are closer to the nearest promoters and enhancers for a given interaction count.

## Discussion and conclusion

The ability to simulate high throughput data with high fidelity to those from real biological experiments is fundamental for benchmarking methods for significant interaction calling analysis and other investigations related to understanding the 3D genome, and ChIA-Sim is a software tool proposed for such a purpose. It imitates, step-by-step, the experimental procedure that produces real specific-protein mediated chromatin interaction data. We demonstrated that one can extract salient features from a real dataset as input to ChIA-Sim, and produce *in-silio* protein-mediated chromatin interaction data that has high fidelity to the real data in the important features. Such data now possess the “ground truth,” a feature that is indispensable for fully benchmarking statistical analysis methods. Although many experimental datasets are available and easily accessible in the public domains and databases, the absolute “true positives” and “false positives” are usually unknown. In the literature, when simulation studies for SIC analyses were performed, they typically just drew data from some known distribution, such as Poisson, which is unrealistic. Therefore, we believe ChIA-Sim has the benefit of generating in silico data with high fidelity to real data and having known ground truth. In some way, it bridges the gap between real experimental data and simulation data from a simple distribution.

As an illustration of an usage of data generated from ChIA-Sim, we carried out a comprehensive evaluation of several SIC analysis methods, which yielded a wealth of information on their operational characteristics. Similar evaluation can be performed to benchmark other analysis methods, if one desires. Based on such results, one may identify relatively better performers so that recommendation can be provided to biological researchers. Characteristics underlying the better performers in terms of their utility for biological data may also be ascertained for development of improved methods; this is a particularly important benefit from using ChIA-Sim data based on what we have observed from benchmarking the four SIC analysis methods. Although we can see from the results that MICC and MDM perform considerably better compared to the other two methods, neither is satisfactory: MDM controls the FPR well, but its power is low; on the other hand, MICC has good power but unacceptably large FPR.

Specifically, our further analysis with four experimental dataset from ChIA-PET substantiates the findings about the operational characteristics of the four SIC analysis methods selected for comparison, and cements the utility of the *in-silico* data from ChIA-Sim for identifying potential issues. In particular, it is clearly seen that genomic distance between the two anchors of an interacting pair and their distance to the closest promoter and enhancer play integral roles in chromatin interaction, and their utilization can lead to better detections of truly interacting pairs that are biologically relevant. Inspired by the results seen in the real data analyses (Figs 5 and 6), we drew similar scatterplots and SVM boundaries for the simulation settings (S7 and S12 Figs in S1 File). One can see that, MICC and Mango, which utilize gDist (the genetic distance between the two anchors) have the tendency of only needing a smaller number of counts for a pair with a larger gDist to declare it truly interacting. On the other hand, MDM, which uses aDist (the annotation distance between the pair of anchors and the nearest pair of promoter-enhancer), requires a larger number of interaction counts to declare a pair with a larger aDist to be a true interacting pair. Since CPT does not utilize either of these two types of distances, the results are not affected at all by aDist or gDist. These results are consistent with those seen from the experimental data analyses, further demonstrating that the various model assumptions of the four methods are reflected in the observed relationships between gDist/aDist and interaction counts.

Nevertheless, none of the methods in the current literature, including those reviewed and compared in this paper, take both of these features into consideration in their SIC procedures. This lack of usage of additional and readily available information may be the most likely reason for the less than satisfactory performance of the methods compared. Finally, although we only focus on demonstrating the usage of ChIA-Sim data for benchmarking SIC analysis methods, data generated from ChIA-Sim can also be used for benchmarking statistical analysis methods for investigating other aspects of 3D chromosomal interactions.

## Supporting information

S1 File (PDF)
