# Supplementary material for: An in silico procedure for generating protein-mediated chromatin interaction data and comparison of significant interaction calling methods
Source: PLoS One. 2024 Jan 17;19(1):e0287521. doi: 10.1371/journal.pone.0287521 (PMC10793909; doi:10.1371/journal.pone.0287521)
Supplement: S1 File — (PDF) [file pone.0287521.s001.pdf]

Supporting information

Table S1. Parameter specifications of settings for demonstrating fidelity to real data

	Pol2			CTCF		
	K562(A)	K562(B)	K562(C)	K562	MCF7	GM
N	879264	879264	879264	91982	195896	171576
N_E	1359	1359	1359	1871	849	969
N_P	1492	1492	1492	2081	932	1000
$N_{\overline{E}}$	100	100	400	100	100	100
$N_{\overline{P}}$	100	100	400	100	100	100
Enzyme	MmeI[a]	HindIII	MmeI[a]	HindIII	HindIII	HindIII

[a] Mmel has two patterns, but only one is used in the simulation study.

Table S2. Parameter specifications for six simulation settings

	Settings					
	1	2	3	4	5	6
N	1×10^5	3×10^5	6×10^5	1×10^6	1×10^6	1×10^6
N_E	400	400	400	400	1000	1500
N_P	400	400	400	400	1000	500
$N_{\overline{E}}$	400	400	400	400	1000	400
$N_{\overline{P}}$	400	400	400	400	1000	400

Table S3. Relative proportions of three types of sites: TFBS, TSS, and non-specific binding sites for several window sizes[a]

TF	Cell line	Window[b]	Enhancer	Promoter	Others
CTCF	MCF7	500/1000	54261	3538	9053
		1000	55005	3537	7492
		2000	54630	4536	5364
		5000	31778	6470	3333
		10000	50650	8406	1998
		20000	47508	10534	921
	K562	500/1000	28669	2680	3961
		1000	29117	2678	3168
		2000	28844	3320	2053
		5000	27796	4613	993
		10000	26139	5970	469
		20000	24074	7460	151
	GM12878	500/1000	65973	5916	29314
		1000	68307	5913	18935
		2000	68512	7026	11444
		5000	67052	8790	3331
		10000	64448	10622	1317
		20000	61028	12634	444
Pol2	K562	500/1000	4884	10460	94472
		1000	5953	10320	88310
		2000	6590	12185	73618
		5000	6946	13905	58893
		10000	7027	14781	44956
		20000	7078	15456	30570

[a] We consider several window sizes since the promoter regions can be from 10-1000 bases [1] to over 20Kb (https://www.genecards.org/cgi-bin/carddisp.pl?gene=LAMA4), whereas the CTCF binding sites can be more than 40Kb from a gene that it regulates [2]. We note that the distribution for the transcription factor Pol2 is very different from those for CTCF. Among the three CTCF datasets, the distribution for the GM12878 cell line is also different from the other two.

[b] For each dataset, 500/1000 indicates that the window size is 500 for the transcription factor and 1000 for the promoter. The rest has the same window size for the transcription factor and the promoter.

Table S4. Numbers of types of pairs for several window sizes[a]

TF	Cell line	Window[b]	E × E	E × P	E/P × N	N × N
CTCF	MCF7	500/1000	33821	6991	8305	1381
		1000	35218	7035	7080	1165
		2000	34543	9801	5234	920
		5000	30604	15821	3469	604
		10000	25420	22441	2274	363
		20000	18956	30219	1165	158
	K562	500/1000	16466	4539	3853	446
		1000	17072	4580	3346	306
		2000	16547	6228	2301	228
		5000	14158	9692	1332	122
		10000	10987	13533	704	80
		20000	7181	17810	272	41
	GM12878	500/1000	48238	14319	27053	3197
		1000	58087	14668	18839	1213
		2000	59517	20312	12242	736
		5000	56098	32305	3854	550
		10000	45583	45109	1637	478
		20000	31935	59870	578	424
Pol2	K562	500/1000	48	9273	13272	41951
		1000	164	14299	10646	39435
		2000	234	20706	11678	31926
		5000	464	26263	11205	26612
		10000	924	30486	7560	25574
		20000	1215	35791	4146	23392

[a]We consider several window sizes since the promoter regions can be from 10-1000 bases [1] to over 20Kb (https://www.genecards.org/cgi-bin/carddisp.pl?gene=LAMA4), whereas the CTCF binding sites can be more than 40Kb from a gene that it regulates [2]. We note that the distribution for the transcription factor Pol2 is very different from those for CTCF. Among the three CTCF datasets, the distribution for the GM12878 cell line is also different from the other two.

[b]For each dataset, 500/1000 indicates that the window size is 500 for the transcription factor and 1000 for the promoter. The rest has the same window size for the transcription factor and the promoter.

Table S5. Simulation results for all pairs. The average and standard deviation (within parentheses) across five replicates are provided for each setting and each method

Setting	Type	Criteria	CPT	MDM	MICC	Mango
1	Overall	FPR	0.990 (0.012)	0.007 (0.009)	0.453 (0.098)	- (-)
		Power	0.934 (0.029)	0.255 (0.048)	0.747 (0.031)	- (-)
	Intra	FPR	0.989 (0.012)	0.008 (0.009)	0.464 (0.099)	0.227 (0.093)
		Power	0.939 (0.028)	0.275 (0.050)	0.751 (0.030)	0.475 (0.096)
	Inter	FPR	1.000 (0.000)	0.000 (0.000)	0.196 (0.107)	- (-)
		Power	0.883 (0.046)	0.037 (0.029)	0.704 (0.058)	- (-)
2	Overall	FPR	0.970 (0.025)	0.003 (0.005)	0.664 (0.084)	- (-)
		power	0.975 (0.017)	0.338 (0.096)	0.786 (0.030)	- (-)
	Intra	FPR	0.969 (0.025)	0.004 (0.005)	0.676 (0.080)	0.474 (0.119)
		Power	0.977 (0.015)	0.367 (0.103)	0.786 (0.029)	0.637 (0.030)
	Inter	FPR	0.983 (0.031)	0.000 (0.000)	0.483 (0.153)	- (-)
		Power	0.957 (0.035)	0.073 (0.043)	0.788 (0.037)	- (-)
3	Overall	FPR	0.974 (0.008)	0.005 (0.003)	0.775 (0.015)	- (-)
		power	0.986 (0.004)	0.322 (0.085)	0.805 (0.011)	- (-)
	Intra	FPR	0.973 (0.009)	0.005 (0.003)	0.781 (0.018)	0.646 (0.025)
		power	0.987 (0.002)	0.351 (0.090)	0.802 (0.012)	0.678 (0.025)
	Inter	FPR	0.986 (0.007)	0.000 (0.000)	0.692 (0.040)	- (-)
		power	0.981 (0.021)	0.057 (0.040)	0.830 (0.016)	- (-)
4	Overall	FPR	0.984 (0.009)	0.001 (0.001)	0.788 (0.032)	- (-)
		power	0.989 (0.005)	0.471 (0.061)	0.798 (0.010)	- (-)
	Intra	FPR	0.985 (0.008)	0.001 (0.001)	0.795 (0.029)	0.693 (0.048)
		power	0.988 (0.005)	0.504 (0.060)	0.796 (0.011)	0.680 (0.016)
	Inter	FPR	0.974 (0.027)	0.000 (0.000)	0.713 (0.090)	- (-)
		power	0.994 (0.004)	0.182 (0.075)	0.818 (0.047)	- (-)
5	Overall	FPR	0.959 (0.006)	0.007 (0.001)	- (-)	- (-)
		power	0.959 (0.019)	0.182 (0.112)	- (-)	- (-)
	Intra	FPR	0.958 (0.007)	0.008 (0.001)	- (-)	0.484 (0.011)
		power	0.962 (0.018)	0.200 (0.122)	- (-)	0.731 (0.023)
	Inter	FPR	0.979 (0.010)	0.000 (0.000)	- (-)	- (-)
		power	0.928 (0.035)	0.014 (0.023)	- (-)	- (-)
6	Overall	FPR	0.976 (0.002)	0.016 (0.001)	- (-)	- (-)
		power	0.938 (0.003)	0.098 (0.002)	- (-)	- (-)
	Intra	FPR	0.977 (0.002)	0.018 (0.001)	- (-)	0.919 (0.003)
		power	0.943 (0.002)	0.108 (0.003	- (-)	0.839 (0.001)
	Inter	FPR	0.956 (0.019)	0.000 (0.000)	- (-)	- (-)
		power	0.886 (0.013)	0.002 (0.001)	- (-)	- (-)

Table S6. Performance evaluation based on AUC for all pairs

Setting	Replicate	CPT	MDM	MICC	Mango
1	1	0.567	0.923	0.762	0.642
	2	0.629	0.973	0.703	0.611
	3	0.626	0.973	0.711	0.605
	4	0.615	0.961	0.755	0.572
	5	0.612	0.958	0.705	0.598
2	1	0.730	0.961	0.604	0.695
	2	0.646	0.932	0.630	0.619
	3	0.731	0.972	0.602	0.626
	4	0.729	0.973	0.664	0.634
	5	0.707	0.975	0.711	0.629
3	1	0.736	0.962	0.601	0.647
	2	0.696	0.944	0.576	0.610
	3	0.760	0.981	0.595	0.610
	4	0.690	0.954	0.565	0.620
	5	0.710	0.955	0.591	0.628
4	1	0.741	0.955	0.559	0.643
	2	0.738	0.963	0.540	0.609
	3	0.708	0.964	0.570	0.576
	4	0.764	0.981	0.596	0.660
	5	0.754	0.978	0.592	0.608
5	1	0.790	0.961	-	0.679
	2	0.699	0.949	-	0.689
	3	0.697	0.962	-	0.682
	4	0.716	0.961	-	0.703
	5	0.698	0.958	-	0.657
6	1	0.552	0.911	-	0.536
	2	0.549	0.908	-	0.530
	3	0.549	0.908	-	0.535
	4	0.553	0.910	-	0.533
	5	0.555	0.911	-	0.535

Table S7. K562 CTCF: Percent of identified true pairs, pairwise agreement between two methods, and cross-tabulation among all methods[1]

(a) Percent true pairs and pairwise agreement[2]

	CPT	MDM	MICC	True
CPT				100.0
MDM	15.6 (15.6, 0.0)			15.6
MICC	13.6 (13.6, 0.0)	93.2 (11.2, 82.0)		13.6
Mango	94 (94.0, 0.0)	15.9 (12.7, 3.2)	14.0 (10.8, 3.2)	94.0

(b) Cross-tabulation

		MICC	T	T	F	F	
CPT	Mango	MDM	T	F	T	F	Total
T	T		2081	604	1081	19624	23390
T	F		695	0	13	789	1497
F	T		0	0	0	0	0
F	F		0	0	0	0	0
		Total	2776	604	1094	20413	24887

[1]The results with CPT threshold of 0.05 or 0.01 led to the same results.
[2]In (a), the last column ("True") is the percent of identified true pairs by a method, while the two percentages in each pair of parentheses are agreements on true and false, respectively.

Table S8. MCF7 CTCF: Percent of identified true pairs, pairwise agreement between two methods, and cross-tabulation among all methods[1]

(a) Percent true pairs and pairwise agreement[2]

	CPT	MDM	MICC	True
CPT				100.0
MDM	13.9 (13.9, 0.0)			13.9
MICC	16.7 (16.7, 0.0)	73.8 (2.2, 71.6)		16.7
Mango	98.1 (98.1, 0.0)	15.3 (13.6, 1.6)	14.8 (14.8, 0.0)	98.1

(b) Cross-tabulation

		MICC	T	T	F	F	
CPT	Mango	MDM	T	F	T	F	Total
T	T		932	6076	5510	33819	46337
T	F		123	774	0	0	897
F	T		0	0	0	0	0
F	F		0	0	0	0	0
		Total	1055	6850	5510	33819	47234

[1]The results with CPT threshold of 0.05 or 0.01 led to the same results.
[2]The last column ("True") is the percent of identified true pairs by a method, while the two percentages in each pair of parentheses are agreements on true and false, respectively.

Table S9. GM12878 CTCF: Percent of identified true pairs, pairwise agreement between two methods, and cross-tabulation among all methods[1]

(a) Percent true pairs and pairwise agreement[2]

	CPT	MDM	MICC	True
CPT				100
MDM	20.3 (20.3, 0)			20.3
MICC	35.3 (35.3, 0)	84.9 (20.2, 64.7)		35.3
Mango	100 (100, 0)	20.3 (20.3, 0)	35.3 (35.3, 0)	100

(b) Cross-tabulation

		MICC	T	T	F	F	
CPT	Mango	MDM	T	F	T	F	Total
T	T		18756	13995	55	60001	92807
T	F		0	0	0	0	0
F	T		0	0	0	0	0
F	F		0	0	0	0	0
		Total	18756	13995	55	60001	92807

[1] The results with CPT threshold of 0.05 or 0.01 led to the same results.
[2] The last column ("True") is the percent of identified true pairs by a method, while the two percentages in each pair of parentheses are agreements on true and false, respectively.

References

1. Le N, Yapp E, Nagasundaram N, Yeh H. Classifying Promoters by Interpreting the Hidden Information of DNA Sequences via Deep Learning and Combination of Continuous FastText N-Grams. Front Bioeng Biotechnol. 2019;7:305.

2. Kim T, Abdullaev Z, Smith A, Ching K, Loukinov D, Green R, et al. Analysis of the vertebrate insulator protein CTCF-binding sites in the human genome. Cell. 2007;128:1231–1245.

(a) K562 Pol2 Scenario B

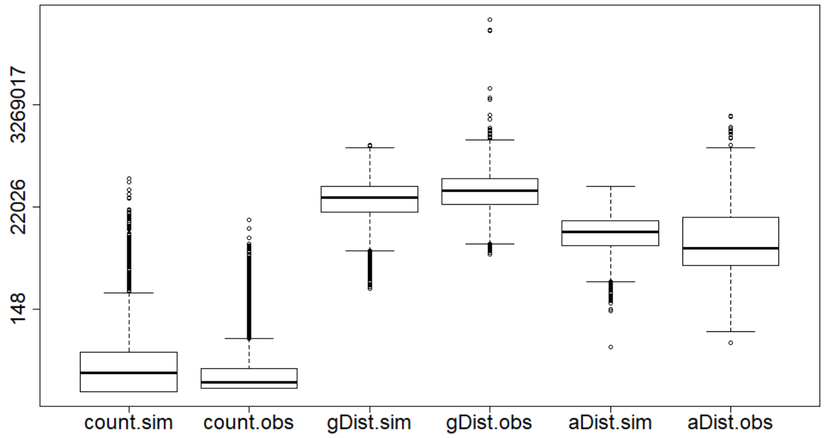

(b) K562 Pol2 Scenario C

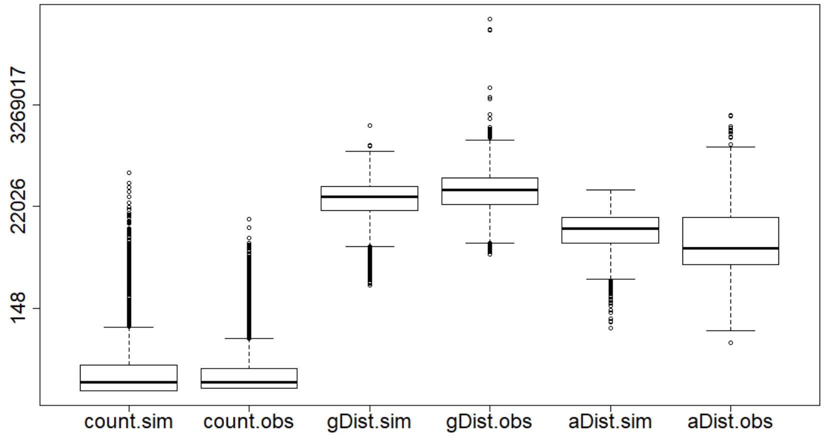

Figure S1. Boxplots comparing several characteristics of data generated from ChIA-Sim with those of the real data based on which the simulation was modeled. The y-axis is marked on the log-scale: the first two boxplots denotes the interaction counts; the last four boxes denote two types of genomic distances as described in the paper. The labels with .obs indicate observed characteristics in the real K562 Pol2 data, whereas those with .sim presents the corresponding characteristics in the data generated from ChIA-Sim. (a) Scenario B, (b) Scenario C; the specific parameter settings are provided in Table S1.

(a) MCF7 CTCF

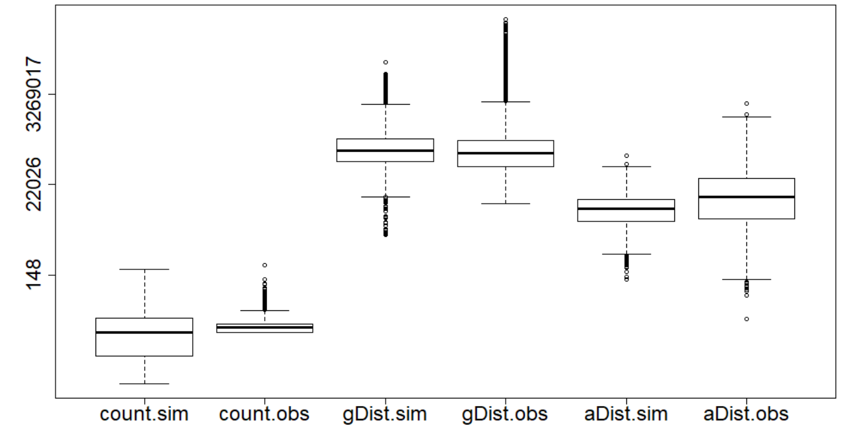

(b) K562 CTCF

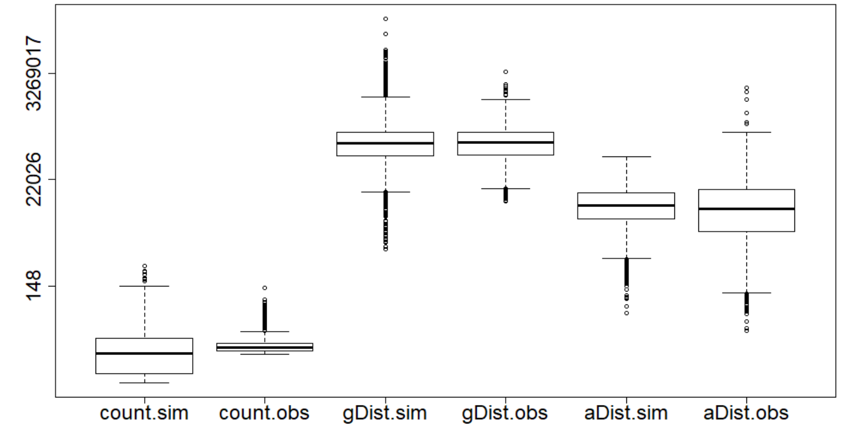

Figure S2. Boxplots comparing several characteristics of data generated from ChIA-Sim with those of the real data based on which the simulation was modeled, for two real datasets. The y-axis is marked on the log-scale: the first two boxplots denotes the interaction counts; the last four boxes denote two types of genomic distances as described in the paper. The labels with .obs indicate observed characteristics, whereas those with .sim presents the corresponding characteristics in the data generated from ChIA-Sim. (a) MCF7 CTCF, (b) K562 CTCF; the specific parameter settings are provided in Table S1.

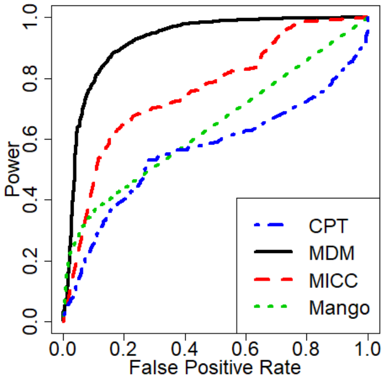

(a) Setting 1

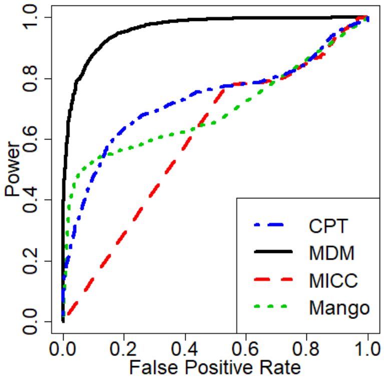

(b) Setting 2

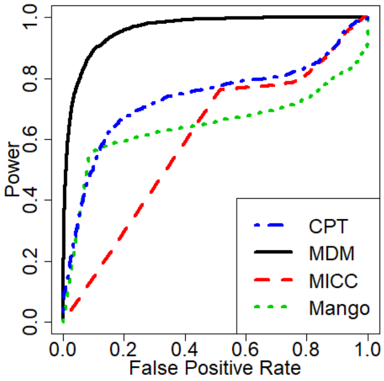

(c) Setting 3

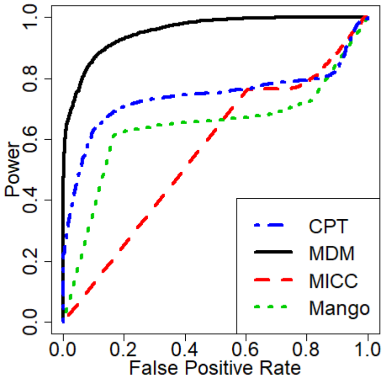

(d) Setting 4

Figure S3. Receiver operating characteristic (ROC) curves for comparing the methods: replicate 1 all pairs.

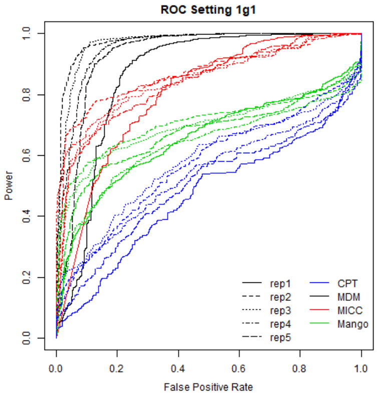

(a)

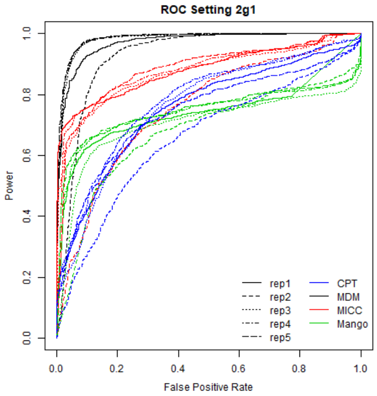

(b)

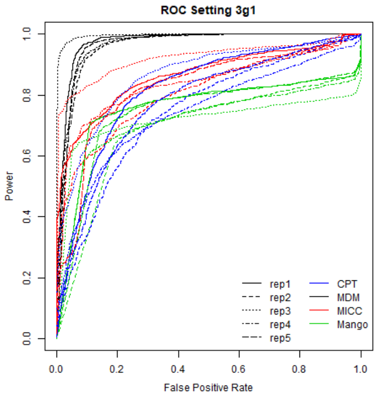

(c)

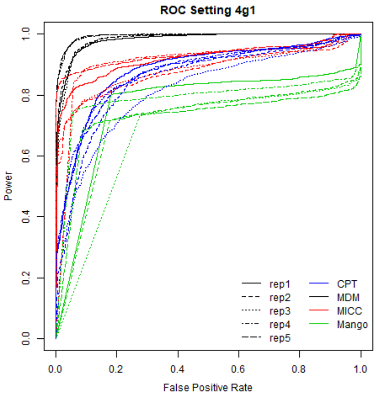

(d)

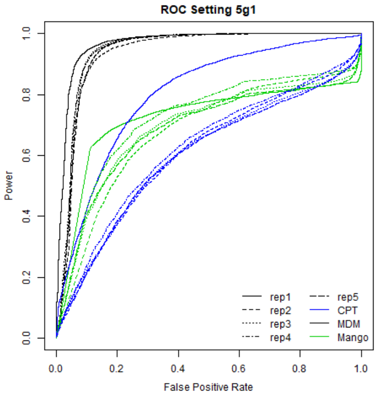

(e)

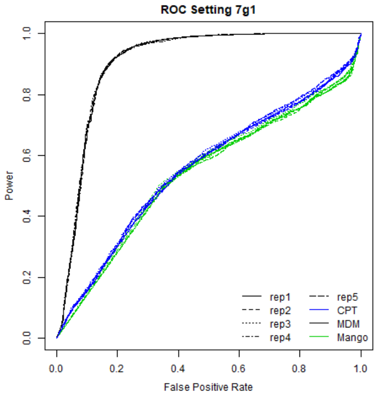

(f)

Figure S4. Receiver operating characteristic (ROC) curves for g1 pairs; shown are results for all five replicates. No results of MICC for setting 5g1 and 6g1 due to errors when running the software.

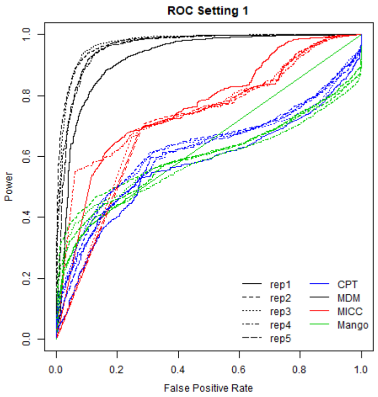

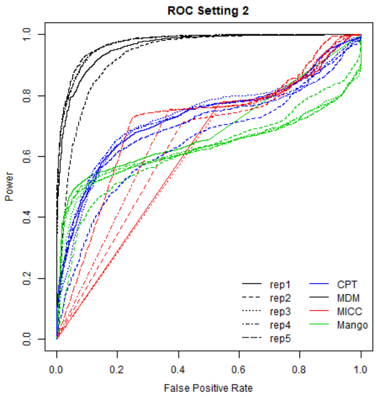

(a) (b)

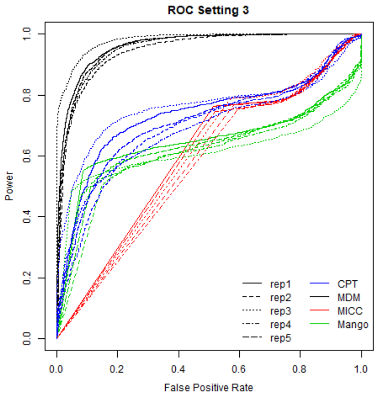

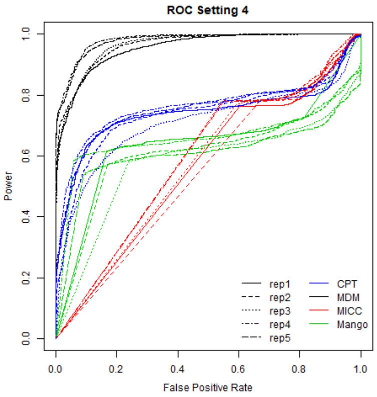

(c) (d)

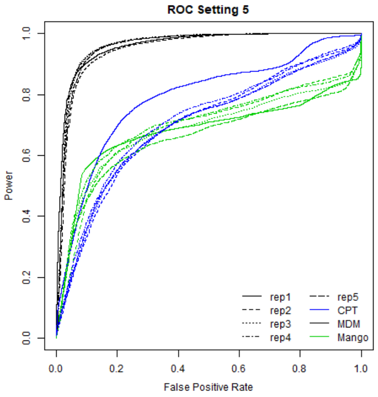

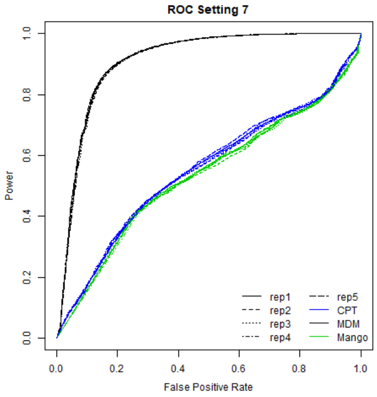

(e) (f)

Figure S5. Receiver operating characteristic (ROC) curves for all pairs; shown are results for all five replicates. No results of MICC for settings 5 and 6 due to errors when running the software.

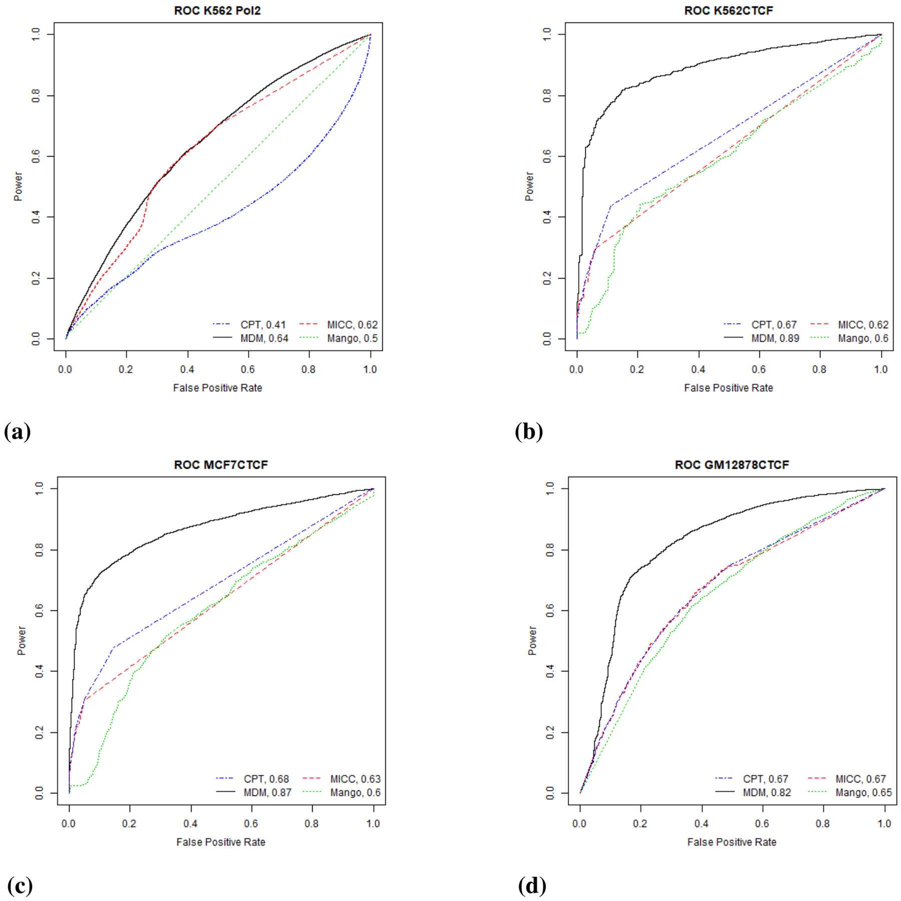

Figure S6. Receiver operating characteristic (ROC) curves comparing the methods for four data sets. (a) K562 Pol2; (b) K562 CTCF; (c) MCF7 CTCF; (d) GM12878 CTCF.

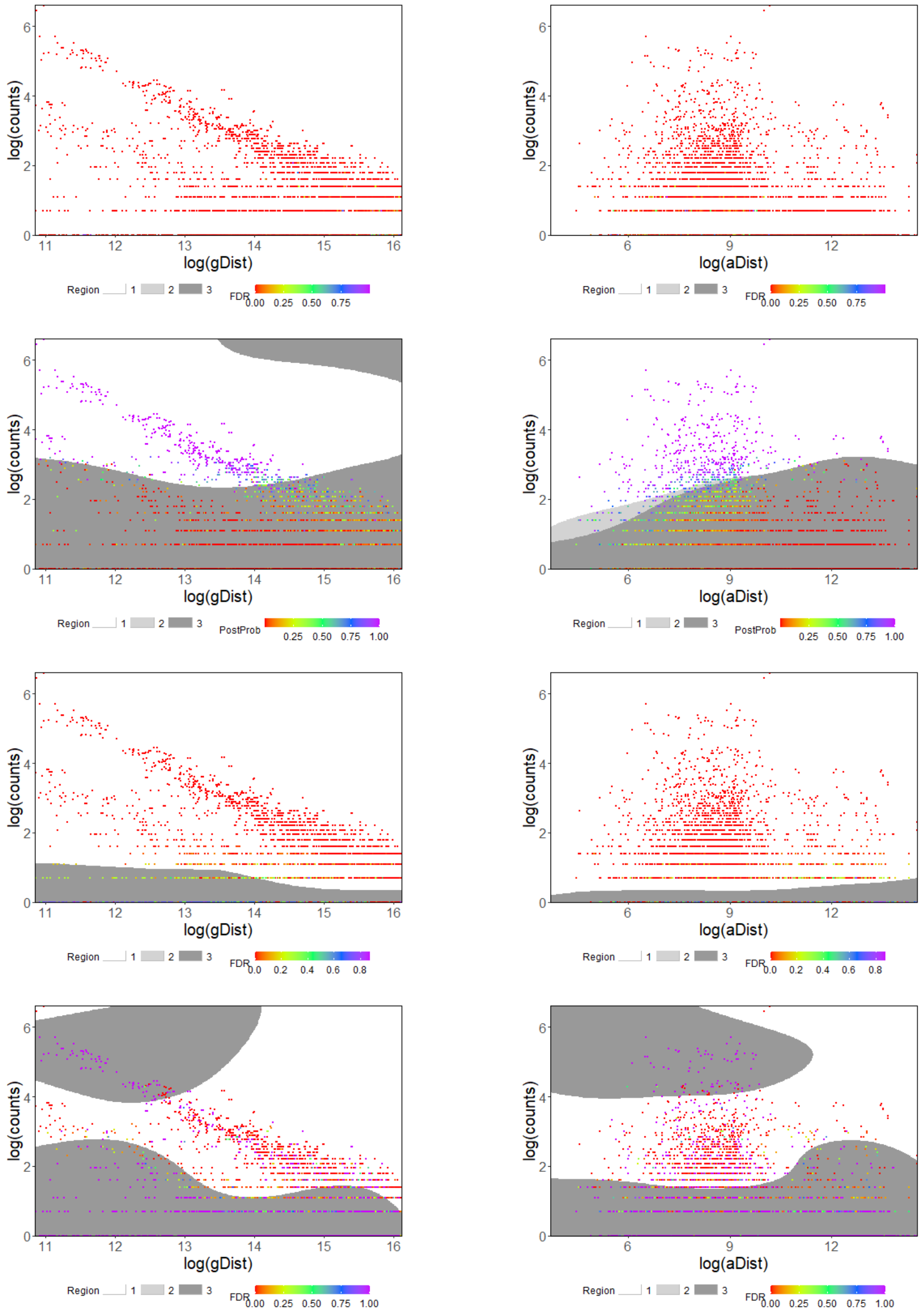

Figure S7. Scatterplots of log(gDist) vs. log(count) (first column) and log(aDist) vs. log(count) for simulation setting 1, with the color gradient of the dots indicating FDR (for MICC, Mango, and CTP) or posterior probability (PP, for MDM). Points in each of the plot are divided into three groups: (1) significant — FDR (0, 0.05) or PP (0.8, 1); (2) moderately- significant — FDR (0.05, 0.2) or PP (0.5, 0.8); (3) non-significant — FDR (0.2, 1) or PP (0, 0.5). The decision boundaries are generated by support vector machine using the R-package e1071, leading to regions shaded in white (Region 1: significant), light-grey (Region 2: moderately significant), and dark grey (Region 3: non-significant), respectively. Row 1: CPT; Row 2: MDM; row 3: MICC; Row 4: Mango.

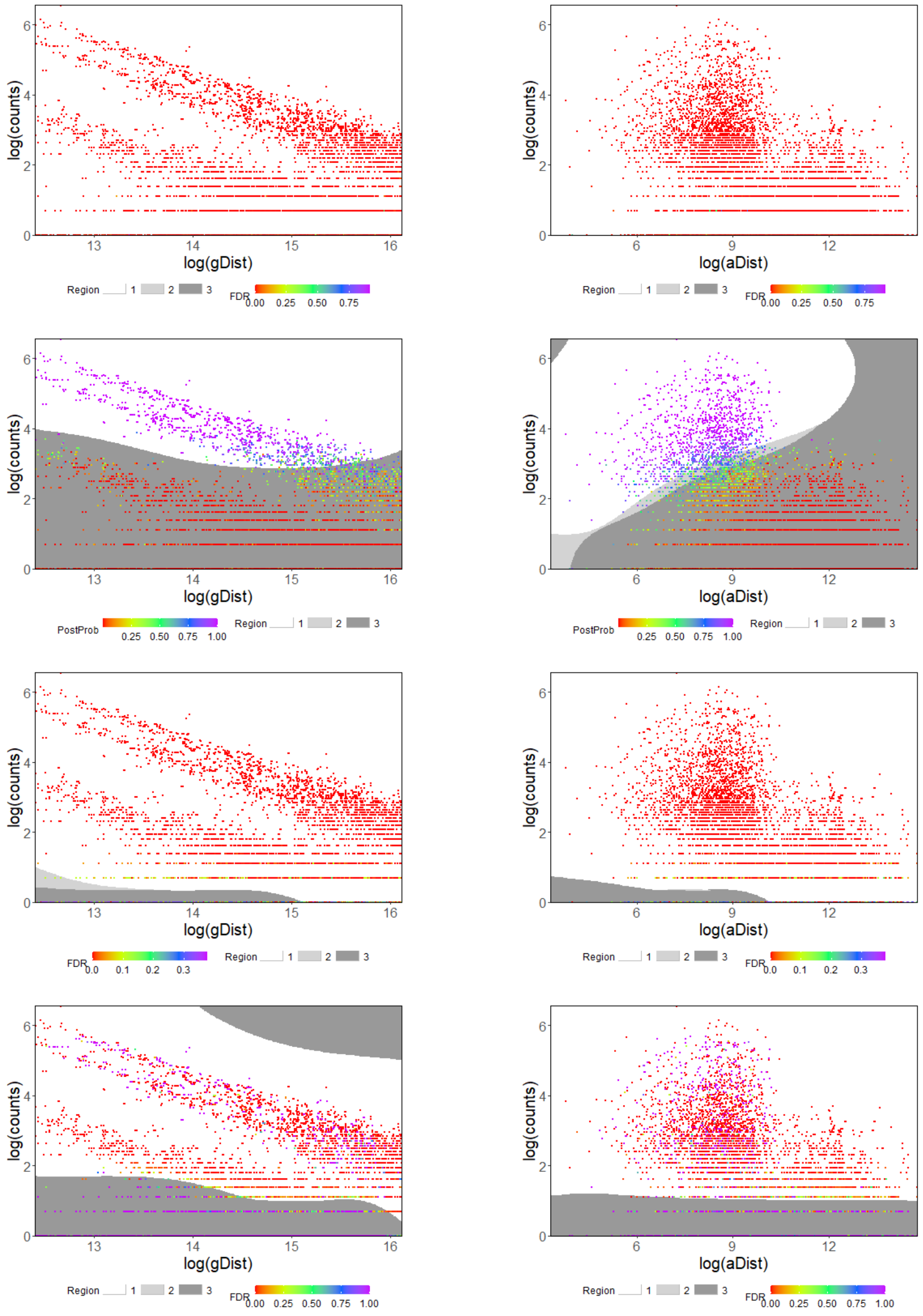

Figure S8. Scatterplots of log(gDist) vs. log(count) (first column) and log(aDist) vs. log(count) for simulation setting 2, with the color gradient of the dots indicating FDR (for MICC, Mango, and CTP) or posterior probability (PP, for MDM). Points in each of the plot are divided into three groups: (1) significant — FDR (0, 0.05) or PP (0.8, 1); (2) moderately- significant — FDR (0.05, 0.2) or PP (0.5, 0.8); (3) non-significant — FDR (0.2, 1) or PP (0, 0.5). The decision boundaries are generated by support vector machine using the R-package e1071, leading to regions shaded in white (Region 1: significant), light-grey (Region 2: moderately significant), and dark grey (Region 3: non-significant), respectively. Row 1: CPT; Row 2: MDM; row 3: MICC; Row 4: Mango.

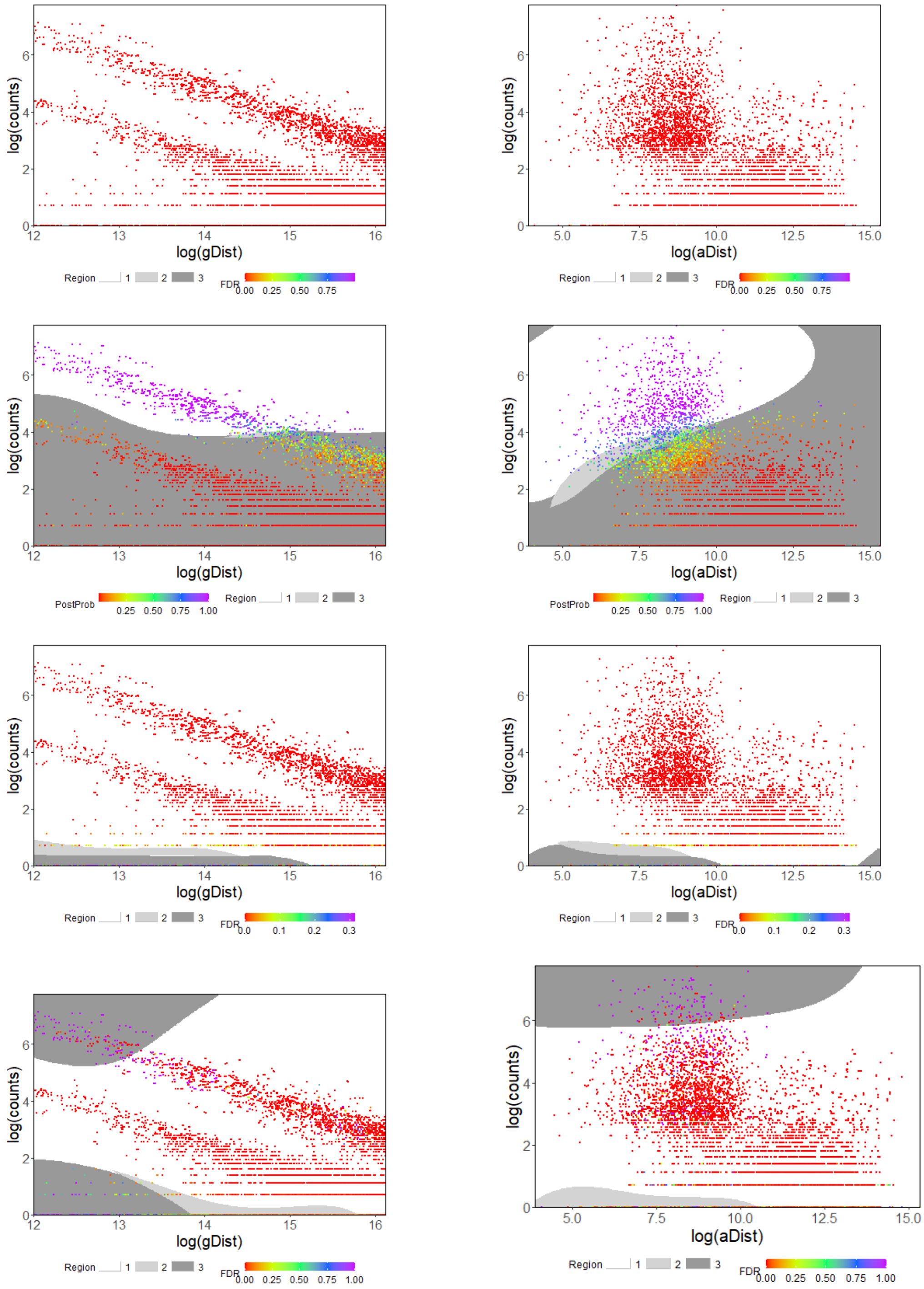

Figure S9. Scatterplots of log(gDist) vs. log(count) (first column) and log(aDist) vs. log(count) for simulation setting 3, with the color gradient of the dots indicating FDR (for MICC, Mango, and CTP) or posterior probability (PP, for MDM). Points in each of the plot are divided into three groups: (1) significant — FDR (0, 0.05) or PP (0.8, 1); (2) moderately- significant — FDR (0.05, 0.2) or PP (0.5, 0.8); (3) non-significant — FDR (0.2, 1) or PP (0, 0.5). The decision boundaries are generated by support vector machine using the R-package e1071, leading to regions shaded in white (Region 1: significant), light-grey (Region 2: moderately significant), and dark grey (Region 3: non-significant), respectively. Row 1: CPT; Row 2: MDM; row 3: MICC; Row 4: Mango.

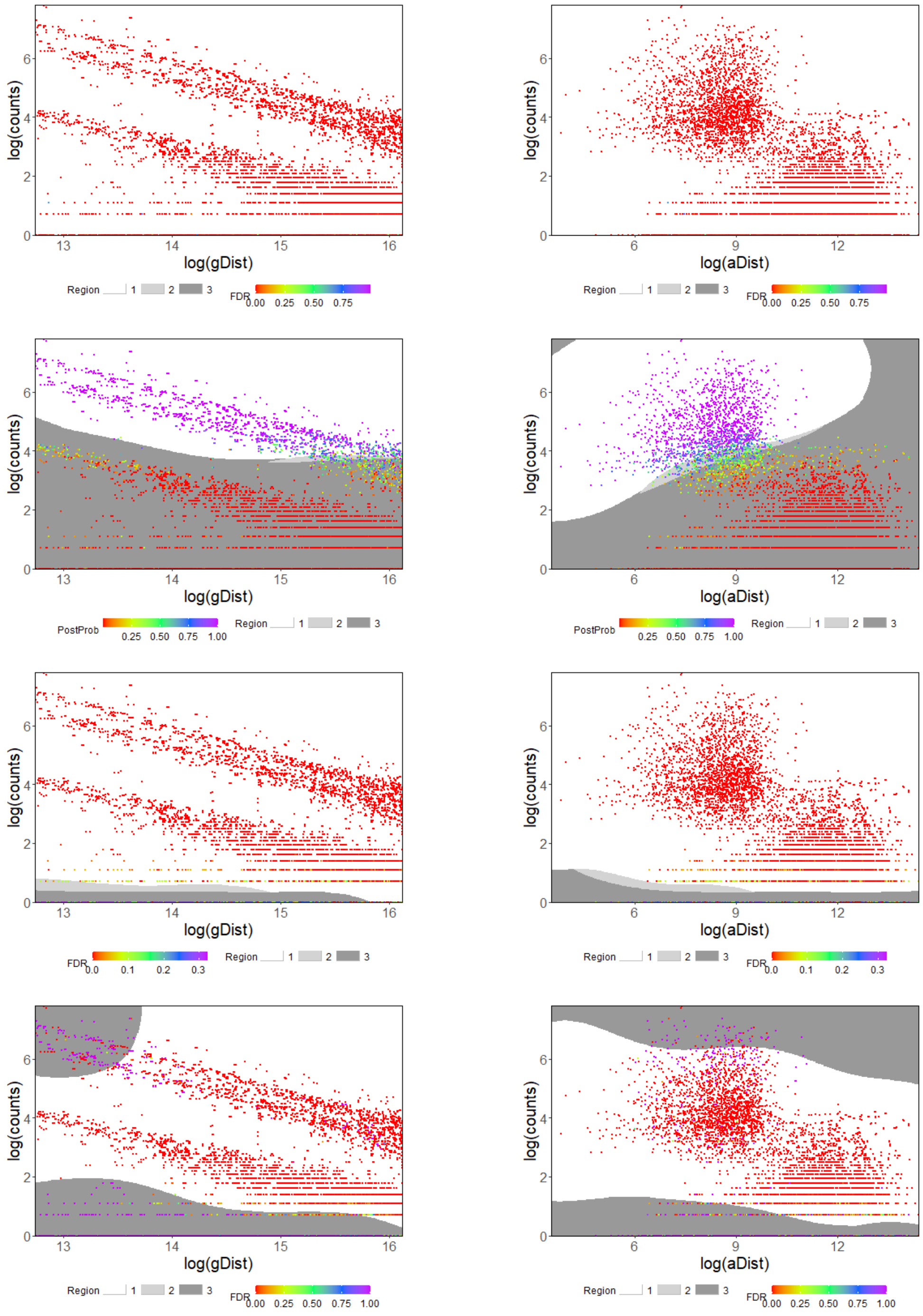

Figure S10. Scatterplots of log(gDist) vs. log(count) (first column) and log(aDist) vs. log(count) for simulation setting 4, with the color gradient of the dots indicating FDR (for MICC, Mango, and CTP) or posterior probability (PP, for MDM). Points in each of the plot are divided into three groups: (1) significant — FDR (0, 0.05) or PP (0.8, 1); (2) moderately-significant — FDR (0.05, 0.2) or PP (0.5, 0.8); (3) non-significant — FDR (0.2, 1) or PP (0, 0.5). The decision boundaries are generated by support vector machine using the R-package e1071, leading to regions shaded in white (Region 1: significant), light-grey (Region 2: moderately significant), and dark grey (Region 3: non-significant), respectively. Row 1: CPT; Row 2: MDM; row 3: MICC; Row 4: Mango.

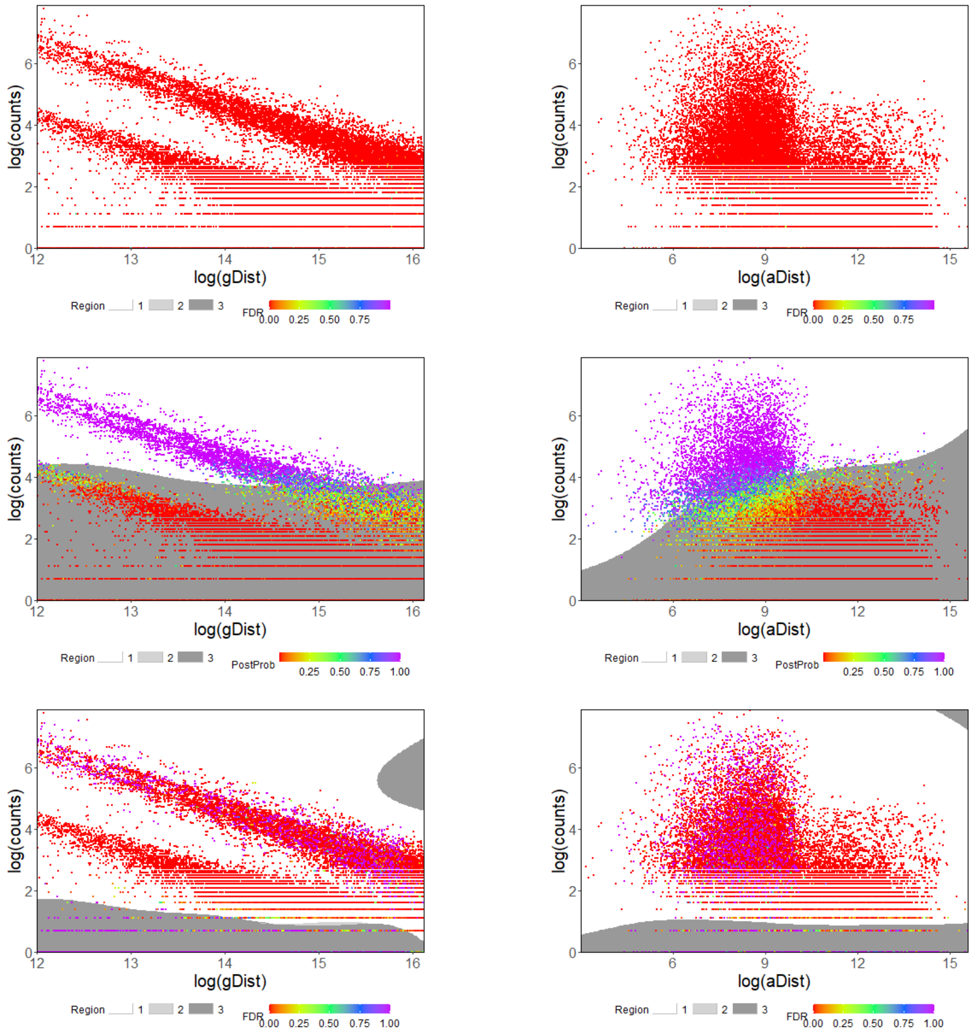

Figure S11. Scatterplot of log(gDist) vs. log(count) (first column) and log(aDist) vs. log(count) for simulation setting 5, with the color gradient of the dots indicating FDR (for MICC, Mango, and CTP) or posterior probability (PP, for MDM). Points in each of the plot are divided into three groups: (1) significant — FDR (0, 0.05) or PP (0.8, 1); (2) moderately- significant — FDR (0.05, 0.2) or PP (0.5, 0.8); (3) non-significant — FDR (0.2, 1) or PP (0, 0.5). The decision boundaries are generated by support vector machine using the R-package e1071, leading to regions shaded in white (Region 1: significant), light-grey (Region 2: moderately significant), and dark grey (Region 3: non-significant), respectively. Row 1: CPT; Row 2: MDM; row 3: MICC; Row 4: Mango.

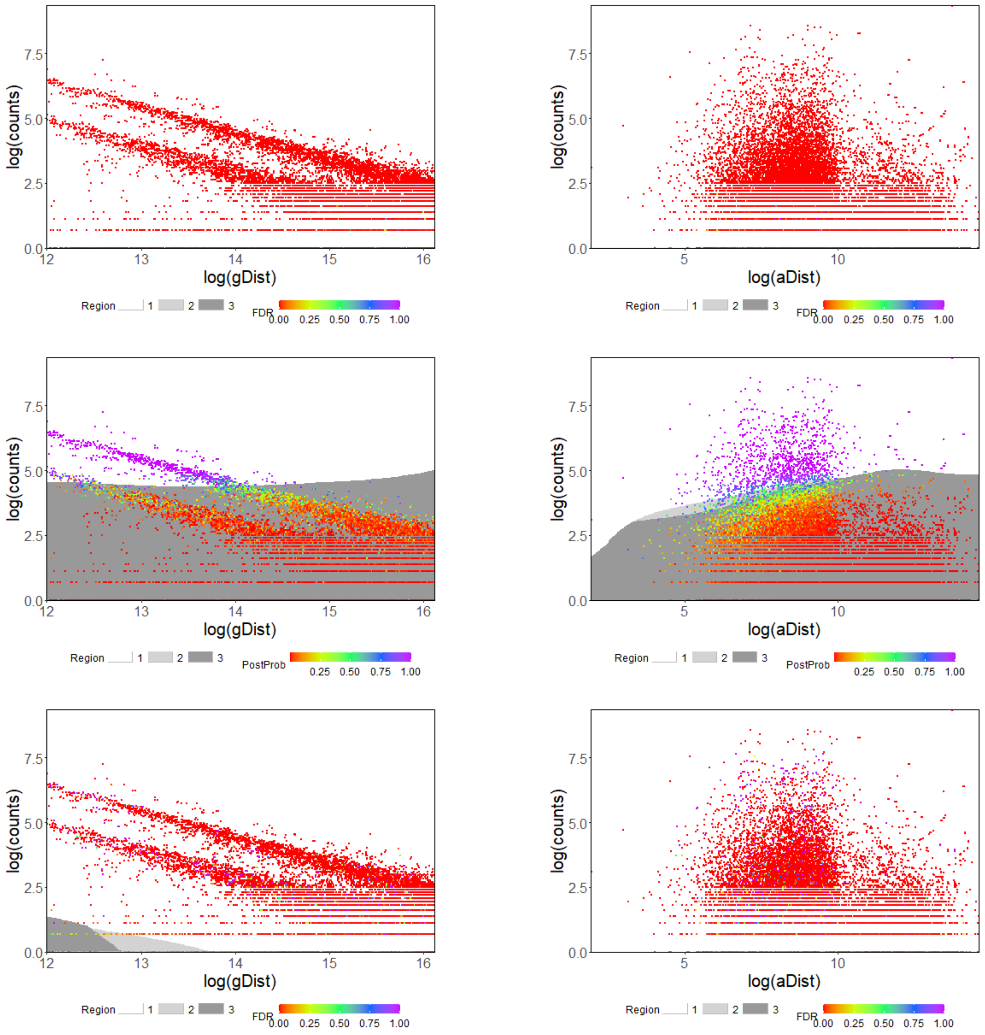

Figure S12. Scatterplots of log(gDist) vs. log(count) (first column) and log(aDist) vs. log(count) for simulation setting 6, with the color gradient of the dots indicating FDR (for MICC, Mango, and CTP) or posterior probability (PP, for MDM). Points in each of the plot are divided into three groups: (1) significant — FDR (0, 0.05) or PP (0.8, 1); (2) moderately-significant — FDR (0.05, 0.2) or PP (0.5, 0.8); (3) non-significant — FDR (0.2, 1) or PP (0, 0.5). The decision boundaries are generated by support vector machine using the R-package e1071, leading to regions shaded in white (Region 1: significant), light-grey (Region 2: moderately significant), and dark grey (Region 3: non-significant), respectively. Row 1: CPT; Row 2: MDM; row 3: MICC; Row 4: Mango.
